# Akt3 activation by R-Ras in an endothelial cell enforces quiescence and barrier stability of neighboring endothelial cells via Jagged1

**DOI:** 10.1016/j.celrep.2024.113837

**Published:** 2024-02-24

**Authors:** Jose Luis Herrera, Masanobu Komatsu

**Affiliations:** 1 Cancer and Blood Disorders Institute, Institute for Fundamental Biomedical Research, and Department of Surgery, Johns Hopkins All Children’s Hospital, St. Petersburg, FL 33701, USA; 2 Department of Orthopaedic Surgery, Johns Hopkins University School of Medicine, Baltimore, MD 21287, USA; 3 Lead contact

## Abstract

Communication between adjacent endothelial cells is important for the homeostasis of blood vessels. We show that quiescent endothelial cells use Jagged1 to instruct neighboring endothelial cells to assume a quiescent phenotype and secure the endothelial barrier. This phenotype enforcement by neighboring cells is operated by R-Ras through activation of Akt3, which results in upregulation of a Notch ligand Jagged1 and consequential upregulation of Notch target genes, such as UNC5B, and VE-cadherin accumulation in the neighboring cells. These signaling events lead to the stable interaction between neighboring endothelial cells to continue to fortify juxtacrine signaling via Jagged1-Notch. This mode of intercellular signaling provides a positive feedback regulation of endothelial cell-cell interactions and cellular quiescence required for the stabilization of the endothelium.

## In brief

Herrera and Komatsu demonstrate that quiescent endothelial cells guide the quiescence of adjacent endothelial cells via Jagged1-Notch intercellular crosstalk. This phenotype reinforcement within the neighborhood of endothelial cells provides a feedback regulation mechanism to secure cell-cell communications and cellular quiescence necessary for the development and maintenance of stable blood vessels.

## Graphical Abstract

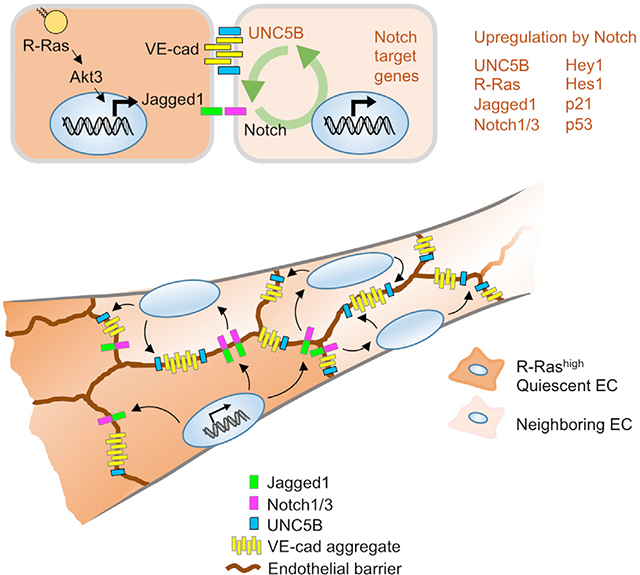

## INTRODUCTION

Intercellular crosstalk between endothelial cells (ECs) is crucial for the regulation of angiogenic sprouting and subsequent maturation/stabilization of new blood vessels as well as for the homeostasis of established blood vessels. Notch and Notch ligands play multiple roles in this regulation. For instance, the Delta-like-4 (Dll4)-Notch signaling between adjacent ECs suppresses tip cell phenotype in the stalk of angiogenic sprouts, and Dll4 deficiency or blockade causes excessive sprouting of immature blood vessels in neonatal mouse retina and in tumors.^1–3^ Jagged1 is another Notch ligand well studied for angiogenesis regulation. A study using a genetic model strongly argues for the pro-angiogenic role of endothelial Jagged1.^4^ Using *pdgfb*-driven Cre to delete endothelial *Jag1*, this study showed that Jagged1 antagonizes Dll4-dependent Notch signaling, leading to increased vessel sprouting and branching during neonatal retinal angiogenesis. However, there are also several other studies suggesting a role for Jagged1 in blood vessel maturation and inhibition of EC sprouting. For instance, the expression of Jagged1 increases as the capillary network begins to mature, and Jagged1 is abundant in the non-angiogenic ECs of arteries and arterioles that are becoming stabilized in postnatal retina.^5^ Notch activation by intraocular injection of Jagged1 peptide suppresses vessel sprouting, branching, and filopodia formation during postnatal retinal angiogenesis.^1^ The upregulation of endogenous Jagged1 in ECs by BMP9-induced ALK1 activation coincides with angiogenesis inhibition in postnatal retina.^6^ Furthermore, Jagged1 peptide suppresses hypersprouting of new blood vessels caused by ALK1 inhibition.^6^ Jagged1 expression by ECs also promotes vascular smooth muscle cell differentiation, thereby contributing to blood vessel stability.^7–12^ EC deletion of *Jag1* causes functional defects in arterial smooth muscle cells and dysfunction of established blood vessels.^13^ Supporting the importance of Jagged1 for vessel integrity, mutations in Jagged1 cause Alagille syndrome,^13^ which exhibits intracranial vessel abnormalities and cerebral aneurysms.^14^ Thus, Jagged1 is not simply a pro-angiogenic Notch ligand that antagonizes the effect of Dll4; this Notch ligand seems to have multifaceted roles in blood vessel formation and maintenance. The role of Jagged1 appears to be complex, and the current literature does not provide a complete picture.

Akt1 upregulates Jagged1 in ECs, which is important for the Notch-dependent survival of vascular smooth muscle cells and vascular stability.^12^ Akt mediates angiogenic stimulus of VEGF via eNOS-dependent nitric oxide production.^15,16^ The global and EC-specific *Akt1*-null mice exhibit impaired angiogenesis in ischemic muscles, highlighting the pro-angiogenic activity of Akt1.^17,18^ On the other hand, a study by another group demonstrated enhanced angiogenic responses associated with impaired vessel maturation and increased permeability in *Akt1*-null mice.^19^ A study of EC-specific *Akt1* deletion in adult mice demonstrated increased capillary density in the heart.^12^ These seemingly contradicting observations of pro- and anti-angiogenic activities of Akt1 may be attributed to the complex multiple roles of Akt in angiogenesis. Another Akt isoform, Akt3, is expressed at a much lower level than Akt1 in ECs. However, despite the low abundance of Akt3 in ECs, this Akt isoform may also play an important but distinct role in EC regulation. A study of endothelial tumors such as infantile hemangioma and angiosarcoma demonstrated that Akt1 promotes, while Akt3 inhibits, the growth of these tumors.^20^ The precise role of Akt3 in angiogenesis and blood vessel regulation is unknown.

Many Ras-family small GTPases activate Akt via PI3-kinase (PI3K). R-Ras is a Ras small GTPase that facilitates endothelial lumenogenesis, blood vessel maturation, and stabilization while suppressing excessive angiogenesis via various mechanisms.^21–23^ It is most abundantly expressed in ECs, pericytes, and vascular smooth muscle cells of mature functional blood vessels as well as in other smooth muscle tissues.^21^ Although it is closely related to K-Ras and H-Ras, R-Ras has little or no cell transforming activity,^24^ which may be due to the lack of ability to activate the Raf-Erk and Ral pathways.^25^ Oncogenic mutations of K-Ras and H-Ras are frequently found in human cancers, but this is not the case for R-Ras.^26^ R-Ras activates PI3K-Akt signaling; however, this axis of Akt signaling appears to be functionally distinct from the canonical pro-angiogenic Akt signaling, because R-Ras activation results in the quiescence of ECs.^21,23^ For instance, R-Ras offsets ECs’ angiogenic response to VEGF by inhibiting ligand-induced VEGF receptor-2 internalization, suppressing downstream signaling.^27,28^ In addition, R-Ras strengthens EC-EC interaction by stabilizing VE-cadherin at the adherens junction, minimizing endothelial permeability.^22^ These activities of R-Ras normalize pathologically regenerating blood vessels.^22,29^ On the other hand, pericyte-targeted *Rras* deletion causes blood-retina-barrier (BRB) breakdown and hypersprouting and branching of the developing capillary plexus in neonatal mouse retina.^30^ The activation of Akt1 and Akt2 by R-Ras promotes lumen formation in sprouting blood vessels (endothelial tubulogenesis) by stabilizing microtubule cytoskeleton via inhibition of GSK-3β.^22^ This pathway is required for producing new patent capillary vessels for the reperfusion of ischemic muscles and the subsequent muscle recovery.^22^ R-Ras also promotes EC survival against Bax-induced apoptosis through Akt activation.^31^ Thus, Akt plays important roles in mediating the effect of R-Ras on ECs.

In this study, we investigated how neighboring ECs cooperate with one another to enforce endothelial integrity and how R-Ras contributes to this crosstalk. We found that activation of Akt, most significantly Akt3 activation, by R-Ras is important for the upregulation of Jagged1, resulting in Notch target gene induction necessary for the quiescence and junctional integrity of neighboring ECs.

## RESULTS

### Endothelial R-Ras activates Notch1/3 through Jagged1 upregulation

**Figure 1.**
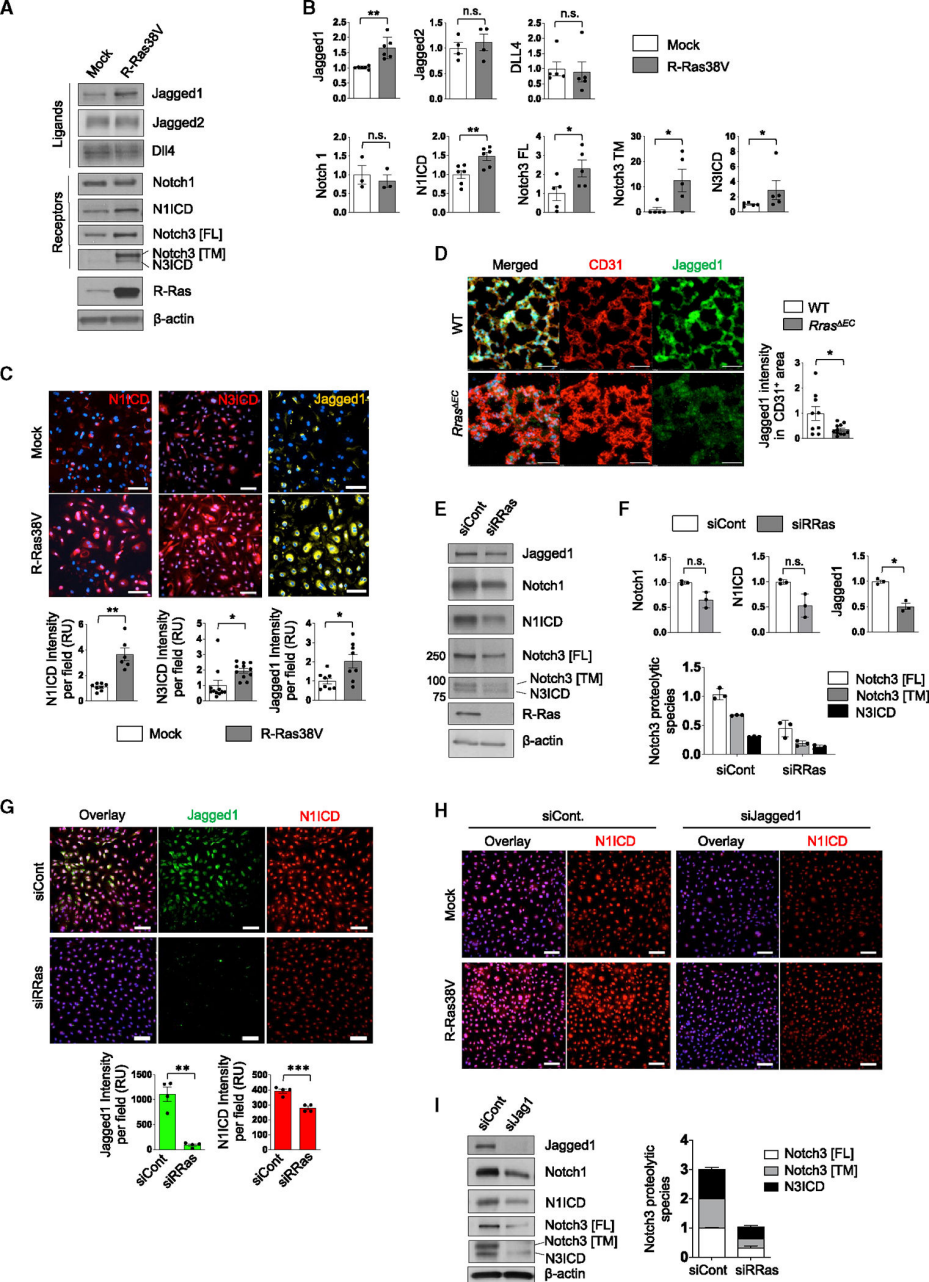
R-Ras upregulates Jagged1 and activates Notch in ECs (A) Western blot analysis of mock- or R-Ras38V-transduced EC lysate from confluent culture. Notch1 intercellular domain (N1ICD) was detected by an antibody specific for it. Since N3ICD-specific antibody is unavailable, it was detected by anti-Notch3 antibody based on the molecular weight of N3ICD. Notch3 [FL], full-length Notch3; Notch3 [TM], transmembrane fragment of cleaved Notch3; N3ICD, Notch3 intercellular domain. (B) Quantification of (A). N = 3 (Notch1), N = 4 (Jagged2), N = 5 (Dll4 and all Notch3 species), N = 6 (Jagged1 and N1ICD). Data are represented as the mean ± SEM. See also Figure S1A. (C) Immunofluorescence staining of confluent EC culture for N1ICD, N3ICD, and Jaggged1 with representative pictures and the quantification of fluorescence intensity presented as relative values (RU). DAPI nuclear staining is blue. Data are represented as the mean ± SEM. N = 3 wells. Pictures of two or three different areas were analyzed for each well. (D) Jagged1 expression in lung capillary ECs was analyzed in *cdh5*-Cre;*Rras*^f/f^ mice (*Rras*^*ΔEC*^) and *Rras*^f/f^ wild-type control mice (WT) by immunostaining. Jagged1 intensity within the CD31^+^ area was quantified and normalized to the total CD31^+^ area. N = 3 mice. Pictures of three different areas of each lung were analyzed. Data are represented as the mean ± SEM. Scale bar, 50 μm. (E) Western blot analysis of small interfering RNA (siRNA) control (siCont) and R-Ras-silenced EC (siRRas). See also Figure S1B. (F) Quantification of (E). N = 3. Data are represented as the mean ± SEM. (G) Jagged1 and N1ICD levels in the control or R-Ras-silenced ECs were determined by immunofluorescence. The graphs present the fluorescence intensity as relative values (RU). DAPI nuclear staining is blue. N = 3 wells, one or two pictures were analyzed per well. Data are represented as the mean ± SEM. See also Figures S1C–S1E. (H) Jagged1 was silenced (siJagged1) in the mock control or R-Ras38V-expressing ECs, and the N1ICD levels in these cells were analyzed by immunofluorescence (red). N = 2. (I) Jagged1 was silenced in the confluent culture of parental ECs, and Notch activation was analyzed by western blot. The graph presents Notch3 in relative values. N = 3. Data are represented as the mean ± SEM. *p < 0.05, **p < 0.01, n.s., not significant.

In an effort to determine the molecular mechanism underlying the regulation of ECs by R-Ras, we found a significant upregulation of a Notch ligand, Jagged1, in confluent cultures of ECs upon the expression of constitutively activated R-Ras, R-Ras38V (Figures 1A, 1B, and S1A). The effect of R-Ras on Jagged1 expression was confirmed by immunofluorescence staining of R-Ras38V-transduced ECs (Figure 1C) as well as by silencing the endogenous R-Ras (Figures 1E and S1B). The regulation of endothelial Jagged1 by R-Ras was also demonstrated *in vivo* using a mouse model of EC-specific *Rras* gene ablation, *cdh5*-Cre;*Rras*^f/f^ (*Rras*^*ΔEC*^) mice. These mice exhibited strong downregulation of Jagged1 in the endothelium of lung capillary vessels as demonstrated by immunostaining (Figures 1D and S1C) as well as by western blot of lung tissue (Figure S1D).

R-Ras did not upregulate the other Notch ligands Jagged2 or Dll4 (Figures 1A and 1B). We observed that the mRNA and protein levels of Notch1 were unaltered by R-Ras38V (Figures 1A, 1B, and S1A). However, the cleaved Notch1 intracellular domain (N1ICD) was significantly increased by R-Ras38V and decreased by silencing endogenous R-Ras, demonstrating that R-Ras regulates Notch1 activity (Figures 1A–1C, 1E–1G, and S1E). We confirmed that this Notch activation is γ-secretase dependent (Figure S1F). While the role of Notch1 in EC stability has been extensively studied, little information is available about the role of Notch3 in EC regulation. We observed a marked increase in the total Notch3 as well as the Notch3 intracellular domain (N3ICD) by R-Ras38V expression while observing the opposite effect by silencing endogenous R-Ras (Figures 1A–1C, 1E, 1F, and S1B). Corroborating these results, strong nuclear accumulation of N1ICD and N3ICD was observed in R-Ras38V-expressing ECs (Figure 1C), and the endothelium of developing retina exhibited a significant decrease in Notch3 expression in *Rras*^*ΔEC*^ mice (Figure S1G). The Notch activation by R-Ras was Jagged1 dependent, as the Jagged1 silencing abrogated the R-Ras-induced increase in N1ICD (Figure 1H). We also showed in parental ECs that Jagged1 silencing decreases protein levels of total Notch1 and Notch3 as well as N1ICD and N3ICD (Figure 1I). These results demonstrate that R-Ras elevates Notch signaling in ECs, in which Jagged1 plays an essential role. Interestingly, we also found a dose-dependent downregulation of endogenous R-Ras by γ-secretase, suggesting that R-Ras expression itself is a subject of Notch regulation (Figure S1H).

### R-Ras signaling in an EC upregulates Hey1, p21, and p53 in adjacent ECs

**Figure 2.**
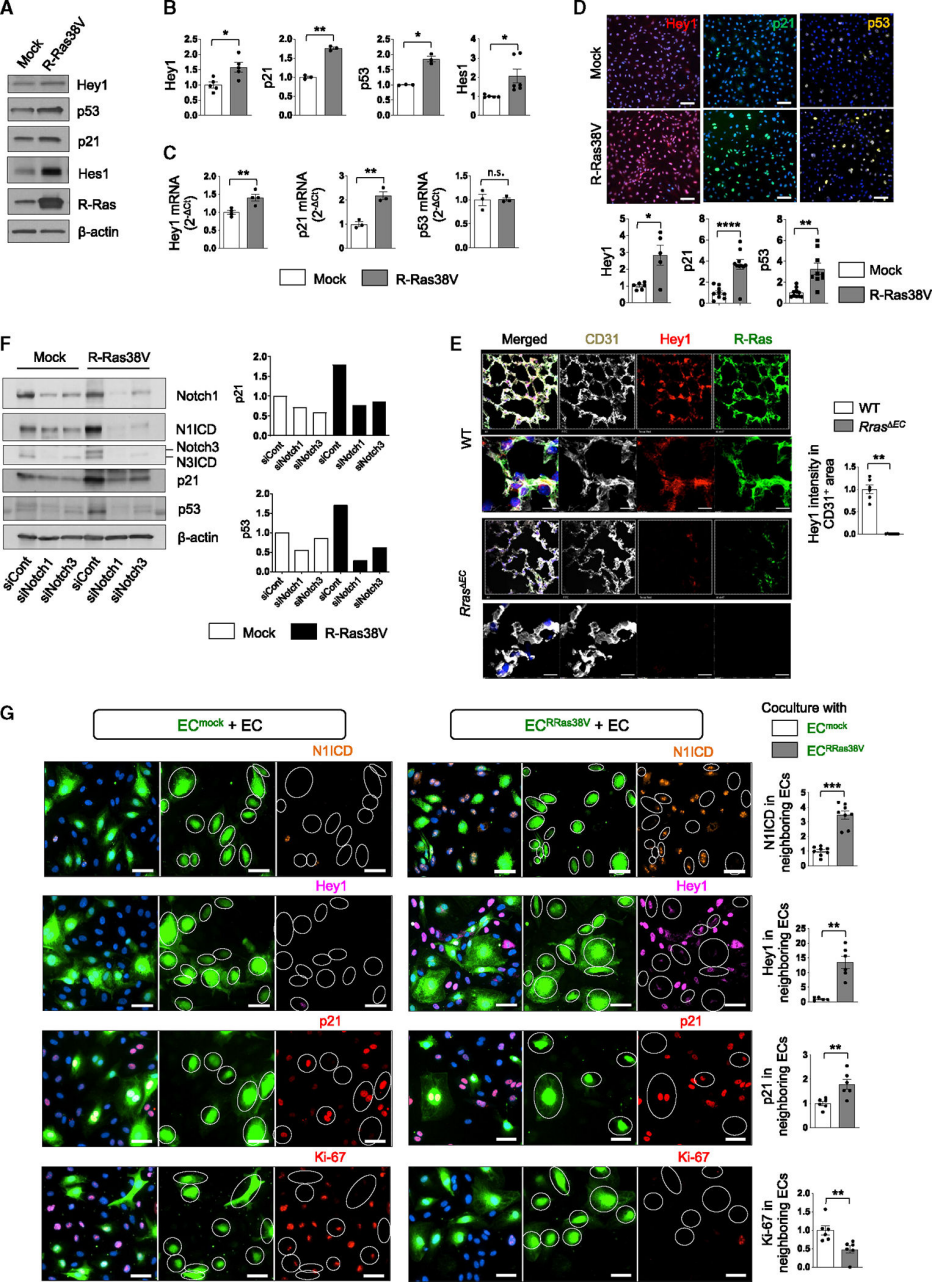
R-Ras upregulates Hey1, p21, and p53 via Jagged1-Notch and inhibits cell cycle of neighboring cells (A) Western blot analysis of mock or R-Ras38V-expressing EC lysates from confluent culture. (B) Quantification of (A). N = 3 (p21 and p53), N = 5 (Hey1), N = 6 (Hes1). Data are represented as the mean ± SEM. *p < 0.05, **p < 0.01, n.s., not significant. (C) RT-qPCR analyses of mRNA expression in relative values (2^−ΔCt^). N 3 or 4. Data are represented as the mean ± SEM. *p < 0.05, **p < 0.01, n.s., not significant. See also Figure S2A. (D) Immunofluorescence and quantification of the relative fluorescence intensity. N = 3 wells, two or three pictures were analyzed for each well. Data are represented as the mean ± SEM. (E) Hey1 and R-Ras expression in lung capillary ECs was analyzed in *cdh5*-Cre;*Rras*^f/f^ mice (*Rras*^*ΔEC*^) and *Rras*^f/f^ wild-type control mice (WT) by immunostaining. Hey1 intensity within the CD31^+^ area was quantified and normalized to the total CD31^+^ area. N = 3 mice; two pictures from each lung were analyzed. Zoomed-in images are 53 × 53 μm. Data are represented as the mean ± SEM. (F) Notch1 or Notch3 was silenced in mock- or R-Ras38V-transduced ECs, and the cell lysate was analyzed by western blot. N = 2. See also Figures S2B–S2D. (G) The mock control (EC^mock^) or R-Ras38V-transduced ECs (EC^RRas38V^) were fluorescently labeled (green) and cocultured with unlabeled, non-transduced parental ECs (EC) at a 1:3 ratio. The cultures were immunostained with the indicated antibodies. The fluorescence intensity in non-transduced ECs was quantified. White circles indicate the positions of EC^mock^ or EC^RRas38V^ cells. N = 2 wells, three or four pictures were analyzed for each well. Data are represented as the mean ± SEM.

To investigate how Jagged1 may contribute to the R-Ras-dependent mechanism of EC quiescence, we studied the effects of R-Ras signaling on genes involved in EC quiescence regulated by Notch, including hairy and enhancer of split-related protein 1 (Hey1), hairy and enhancer of split-1 (Hes1), cyclin-inhibitor p21, and p53.^32–35^ R-Ras38V expression in ECs resulted in the increase of Hey1, Hes1, p21, and p53 protein levels by 50%, >100%, 75%, and 80%, respectively (Figures 2A and 2B). RT-qPCR analyses demonstrated the upregulation of Hey1 and p21 mRNA expression by R-Ras38V, whereas the p53 mRNA level was unaltered, suggesting that p53 is regulated posttranscriptionally or regulated by protein stability (Figures 2C and S2A). Immunofluorescence staining confirmed the upregulation of the three proteins in ECs (Figure 2D). Consistently, Hey1 expression was significantly reduced in the lung capillary endothelium of *Rras*^*ΔEC*^ mice (Figure 2E). To examine whether these effects of R-Ras are dependent on the Notch activation, we treated ECs with γ-secretase inhibitors. Blocking Notch activation with N-[N-(3,5-difluorophenacetyl)-L-alanyl]-s-phenylglycine t-butyl ester (DAPT) or LY411575 abolished N1ICD and suppressed the p21 upregulation by R-Ras in ECs (Figures S2B and S2C). Notch1 or Notch3 knockdown also blocked the R-Ras-dependent upregulation of p21 and p53, further demonstrating the significance of Notch for the R-Ras effects (Figure 2F). Interestingly, silencing either Notch1 or Notch3 resulted in the significant reduction of the other Notch proteins, suggesting a cross-regulation between the two Notch isoforms (Figures 2F and S2D).

To further investigate how R-Ras expressed in an EC affects adjacent ECs through the Jagged1-Notch signaling, we set up a coculture of R-Ras38V-transduced, green fluorescence-labeled ECs (EC^RRas38V^) with unlabeled, non-transduced wild-type ECs (Figure 2G). R-Ras signaling in EC^RRas38V^ resulted in nuclear accumulation of N1ICD in adjacent ECs, indicating that the Notch1 pathway had been activated in the neighboring cells (Figure 2G). The Notch effectors Hey1 and p21 also displayed strong nuclear accumulation in adjacent ECs, indicating that Notch target genes had been induced in these cells (Figure 2G). The mock-transduced fluorescently labeled ECs (EC^mock^) did not have these effects on neighboring ECs (Figure 2G). These effects were reduced when Jagged1 was silenced on R-Ras38V- or mock-transduced cells only (Figure S3). These results demonstrate that the R-Ras-Jagged1-Notch1/3 axis upregulates the key inducers of cell quiescence in neighboring ECs.

### R-Ras in an EC limits proliferation and migration of neighboring ECs

**Figure 3.**
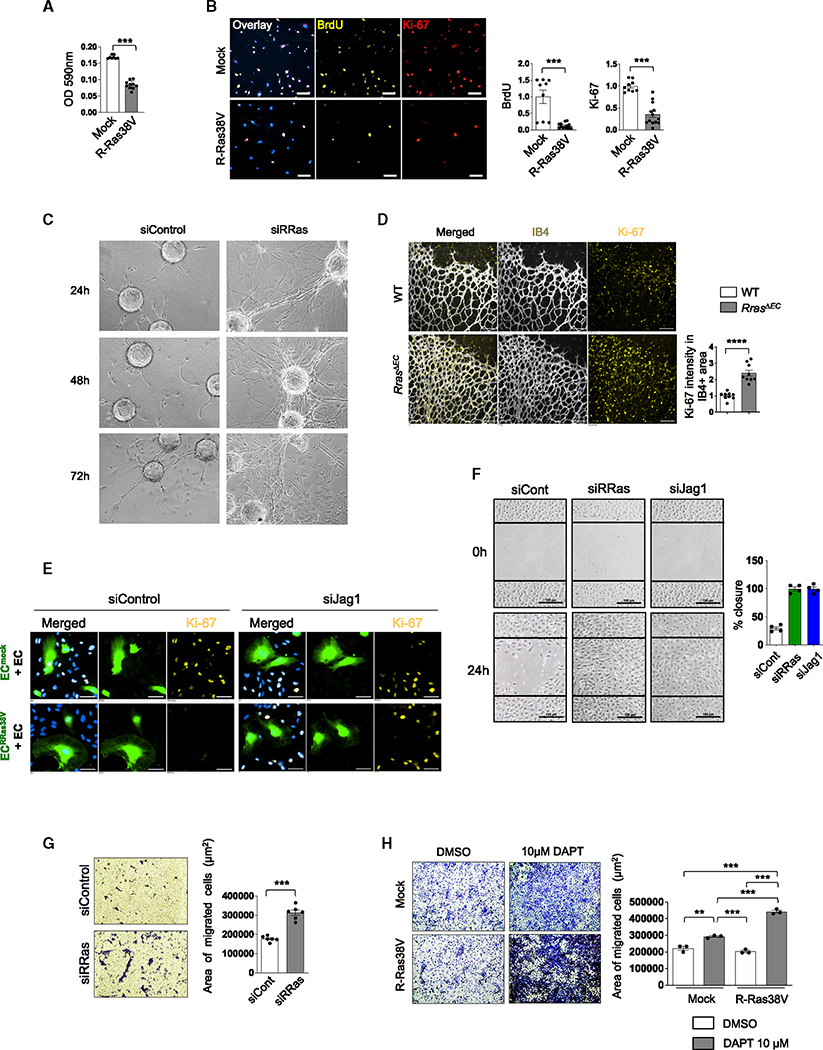
R-Ras inhibits proliferation and migration of ECs (A) MTT assay of mock and R-Ras38V-expressing ECs. N = 10 wells. Data are represented as the mean ± SEM. (B) BrdU incorporation and Ki-67 staining of mock and R-Ras38V-expressing ECs to assess cell cycling. N = 3 wells, three pictures analyzed per well. Data are represented as the mean ± SEM. See also Figure S4. (C) *In vitro* endothelial sprouting assay. Endothelial sprouting of R-Ras-silenced ECs were analyzed in 3D fibrin gel culture at 24, 48, and 72 h. Silencing of R-Ras exacerbated the vessel sprouting. (D) Ki-67 staining of whole-mount neonatal retinal vasculature (day 6). ECs are visualized by IB4 staining. Ki-67 intensity in the IB4^+^ area was quantified and normalized to the IB4^+^ area. N = 4 retinas, two or three pictures were analyzed for each retina. Data are represented as the mean ± SEM. (E) Ki-67 staining of proliferating ECs in the coculture system. Green-labeled mock- (EC^mock^) or R-Ras38V-transduced ECs (EC^RRas38V^) were transfected with *Jag1* (siJag1) or control siRNA for 15 h and then cocultured with non-transduced unlabeled parental ECs (EC) for an additional 48 h. DAPI was used for nuclear staining (blue). (F) Migration of R-Ras- or Jagged1-silenced ECs was analyzed by a scratch-wound assay and quantified as the percentage of closure of the wound by migrated ECs in 24 h. N = 4 culture dishes. Data are represented as the mean ± SEM. (G) Transwell migration assay of the control and R-Ras-silenced ECs. Cells that migrated to the bottom side of the Transwell inserts were stained with 0.5% crystal violet and quantified. N = 6 Transwell inserts. Data are represented as the mean ± SEM. See also Figure S5. (H) Transwell migration of ECs treated with or without 10 μM DAPT. N = 3 Transwell inserts. Data are represented as the mean ± SEM.

These findings prompted us to analyze the effects of R-Ras on EC proliferation. MTT assay showed that R-Ras38V expression decreases EC proliferation approximately by 50% (Figure 3A). Consistently, bromodeoxyuridine (BrdU) incorporation assay and Ki-67 staining demonstrated inhibition of cell-cycle progression by R-Ras (Figures 3B and S4). In agreement with the role of R-Ras as an angiostatic regulator, R-Ras silencing enhanced angiogenic sprouting of ECs in a fibrin-gel 3D culture with exacerbated filopodia formation and vessel branching (Figure 3C). The inhibitory effect of R-Ras on EC proliferation was also demonstrated *in vivo*. Ki-67 immunostaining of the *Rras*^*ΔEC*^ postnatal day 6 retina exhibited a marked increase in EC proliferation compared with the control retinas (Figure 3D). Further studies using the *in vitro* coculture method showed that ECs adjacent to EC^RRas38V^ largely lost immunoreactivity for anti-Ki-67 antibody, demonstrating cell-cycle arrest, whereas ECs adjacent to EC^mock^ showed strong Ki-67 staining, indicating proliferation of these cells (Figure 2G). Importantly, when Jagged1 is silenced in EC^RRas38V^, these ECs failed to arrest the cell cycle of adjacent ECs (Figures 3E and S3). Combined results suggest a mechanism of contact inhibition enforced by R-Ras and transmitted to neighboring ECs via Jagged1.

We next examined the impact of R-Ras signaling on the motility of ECs. R-Ras inhibits EC migration.^21,23^ Thus, R-Ras silencing enhanced migration of ECs in a scratch-wound assay (Figure 3F). Migration assays using Transwell culture inserts confirmed the inhibitory effect of R-Ras on EC motility (Figures 3G and S5). Notably, the silencing of Jagged1 increased EC migration similar to the silencing of R-Ras (Figure 3E). Moreover, Notch inhibition by DAPT increased migration of both mock-transduced ECs and R-Ras38V-expressing ECs (Figure 3H). The effect of Notch inhibition was greater in R-Ras38V-expressing ECs than the control ECs, suggesting that R-Ras enhances, not inhibits, EC migration in the absence of Notch (Figure 3H).

Since cells undergoing senescence are cell-cycle arrested and can exhibit increased expression of p21, p53, or Notch3,^36–38^ we examined whether R-Ras signaling drives ECs into senescence. In an assay of senescence-associated β-galactosidase activity, no β-galactosidase staining was detected in the EC^mock^ or EC^RRas38V^ culture (Figure S6A). Likewise, no difference was found in the expression of p16 or c-Myc, two markers of senescent cells^39^ (Figure S6B). These results show that R-Ras signaling promotes EC quiescence but not senescence.

### R-Ras in an EC enhances barrier function of neighboring ECs through Notch-dependent upregulation of UNC5B

**Figure 4.**
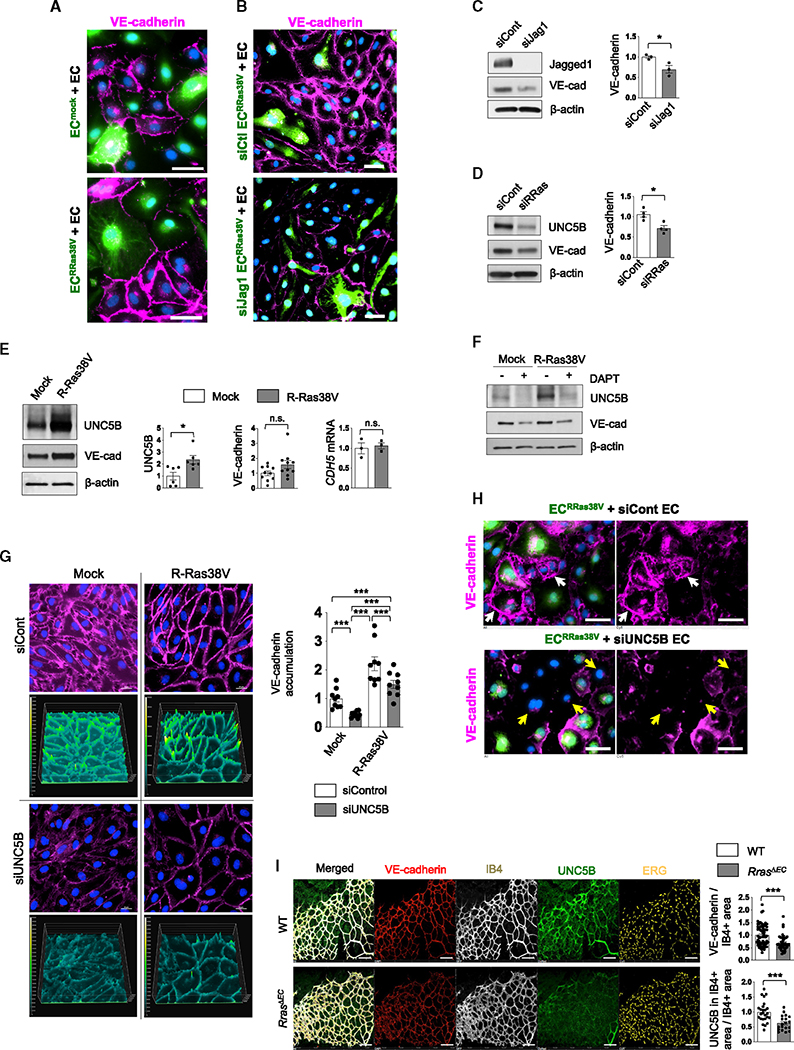
R-Ras-Notch pathway strengthens barrier integrity of neighboring ECs via UNC5b (A) Cocultures of EC^mock^ or EC^RRas38V^ (green) with unlabeled, non-transduced parental ECs (EC) were stained for VE-cadherin to analyze the integrity of the adherens junctions between ECs. DAPI stain is in blue. Scale bars, 10 μm. N = 3. (B) Unlabeled parental ECs were cocultured with green-fluorescence-labeled R-Ras38V-expressing ECs with or without Jagged1 silencing (EC^RRas38V/siJag1^ or EC^RRas38V/siCont^), and the adherens junctions between the ECs were examined by VE-cadherin staining. Scale bars, 50 μm. N = 3. (C and D) Western blot of control and Jagged1-silenced (C) or R-Ras-silenced (D) ECs. VE-cadherin protein levels were quantified and normalized to β-actin. VE-cad, VE-cadherin. Data are represented as the mean ± SEM. N = 3. See also Figures S7A and S7B. (E) The protein levels of UNC5b (N = 6) and protein (N = 10) and mRNA levels (N = 3) of VE-cadherin (CDH5) in mock- or R-Ras38V-transduced ECs were determined by western blot and RT-qPCR. Data are represented as the mean ± SEM. (F) UNC5b and VE-cadherin western blot of mock- or R-Ras38V-transduced ECs treated with or without 10 μM DAPT. N = 3. (G) VE-cadherin immunostaining (magenta) of mock- or R-Ras38V-transduced ECs with or without UNC5b silencing. Surface plot images show the fluorescence intensity of pixels in color scale. Data are represented as the mean ± SEM. N = 3 wells, three pictures analyzed for each. See also Figures S7C–S7F. (H) EC^RRas38V^ (green) were cocultured with UNC5b-silenced or control ECs (unlabeled). The coculture was then stained for VE-cadherin (magenta). Top: white arrows indicate a strong accumulation of VE-cadherin at the cell membrane of control ECs adjacent to EC^RRas38V^. Bottom: yellow arrows indicate the loss of VE-cadherin at the cell-cell junctions in neighboring ECs in which UNC5B was silenced. Representative images of three independent experiments are shown. Scale bars, 10 μm. (I) VE-cadherin and UNC5b staining of whole-mount neonatal retinal vasculature (day 6). ECs are visualized by IB4 staining. VE-cadherin or UNC5b intensity in the IB4^+^ area was quantified and normalized to the IB4^+^ area. N = 8 retinas, five to eight pictures per retina. Data are represented as the mean ± SEM.

Notch is important for the stability of blood vessels. However, the molecular mechanism of the endothelial integrity regulation by Notch has not been fully understood. R-Ras enhances the endothelial barrier function by facilitating VE-cadherin accumulation at the adherens junction as previously demonstrated^23^ and shown here by R-Ras silencing (Figure S7A). In the current study, we investigated the role of Notch in the R-Ras-dependent endothelial barrier formation. The interaction between ECs was analyzed in the coculture consisting of green-fluorescence-labeled EC^RRas38V^ and unlabeled non-transduced ECs. This study revealed strong accumulation of VE-cadherin at the adherens junctions between non-transduced ECs that are in contact with EC^RRas38V^ (Figure 4A), demonstrating that the effect of R-Ras is transmitted to neighboring ECs. This transmission was dependent on the Jagged1-mediated crosstalk, since silencing Jagged1 in EC^RRas38V^ abolished the effect (Figure 4B). We also showed that either Jagged1 or R-Ras silencing reduces VE-cadherin in ECs (Figures 4C, 4D, and S4B).

**Figure 5.**
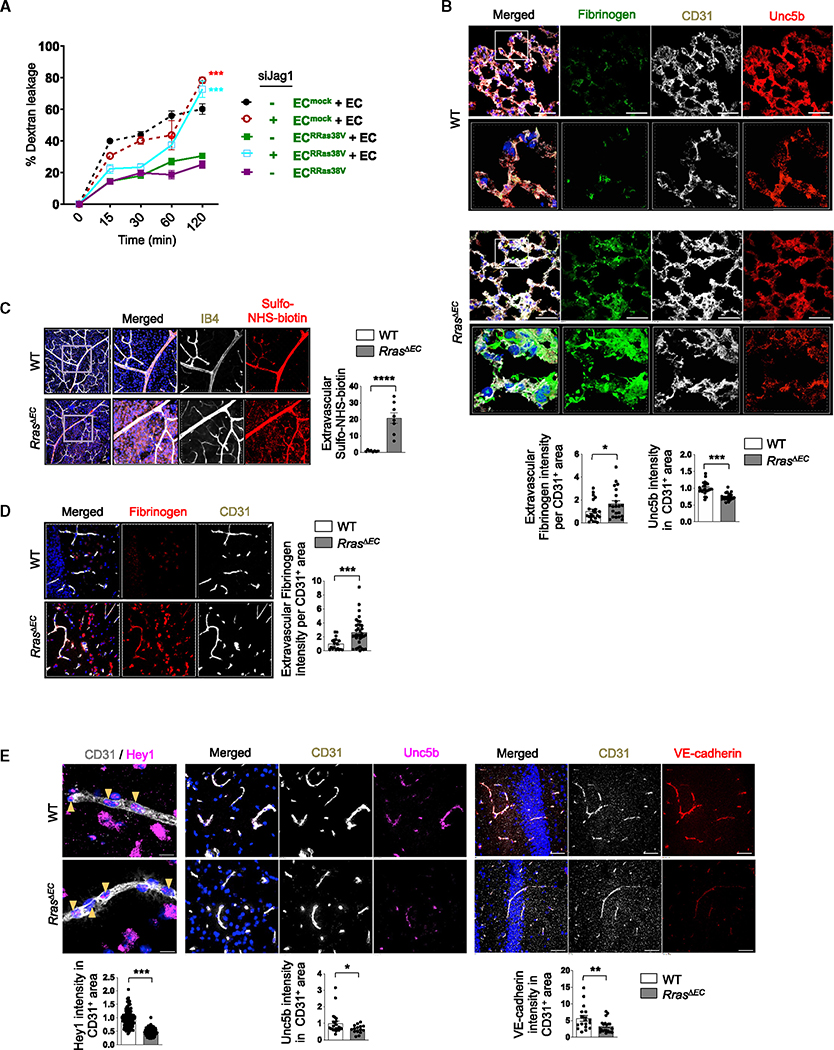
Disruption of Jagged1-Notch and abnormalities of *Rras*^*ΔEC*^ vasculature (A) EC^mock^ or EC^RRas38V^ with or without Jagged1 silencing were cocultured with parental ECs at a 1:3 ratio. Confluent monolayers of various coculture combinations were examined for endothelial permeability by dextran leakage. Single culture of EC^RRas38V^ was included for comparison with the cocultures. N = 3. Data are represented as the mean ± SEM. (B) Extravascular fibrinogen in the lung was analyzed by immunostaining in 3D-reconstructed confocal images. Fluorescence intensity was normalized to the total CD31^+^ area. Unc5b staining intensity within the CD31^+^ area was quantified and normalized to the total CD31^+^ area. The white square insets of 72 × 72 μm are shown in higher magnification in the lower images. N = 3 mice, seven or eight pictures analyzed for each lung. Data are represented as the mean ± SEM. (C) Vascular permeability in adult retina was examined by perfusion with a biotinylation reagent (sulfo-NHS-biotin) followed by detection with streptavidin and IB4 staining. The retinal areas indicated by the squares are magnified on the right. The extravascular sulfo-NHS-biotin fluorescence intensity was quantified from the images and normalized to the total IB4^+^ area. N = 6 retinas. Data are represented as the mean ± SEM. See also Figure S8A. (D) Fibrinogen staining (red) to assess plasma leakage in the hippocampus. The intensity of extravascular fibrinogen was normalized to the total CD31^+^ area. N = 3 mice, six or more pictures analyzed for each brain. Data are represented as the mean ± SEM. (E) Hey1, Unc5b, or VE-cadherin intensity within the CD31^+^ area of the hippocampus was quantified and normalized to the CD31^+^ area. DAPI for nuclear staining is blue. Scale bars, 50 μm. N = 3 mice, five or more pictures analyzed for each brain. Data are represented as the mean ± SEM. See also Figure S8B. *p < 0.05, **p < 0.01, ***p < 0.001, ****p < 0.0001.

UNC5B is a netrin receptor of ECs, which plays an important role in stabilizing endothelial barrier integrity.^40–43^ We found that R-Ras38V upregulates UNC5B in a Notch-dependent manner (Figures 4E and 4F), whereas R-Ras silencing downregulates UNC5B (Figures 4D and S7B). There was also a tendency for VE-cadherin protein increase, while mRNA expression was unaffected (Figure 4E), an observation consistent with VE-cadherin stabilization at adherens junctions (Figure 4A). Notch inhibition downregulated both UNC5B and VE-cadherin (Figure 4F). UNC5B silencing caused diffused and reduced VE-cadherin staining of mock- or R-Ras38V-transduced EC monolayers as well as in non-transduced EC monolayers, indicating a disruption of the adherens junctions (Figures 4G and S7C–S7F). Moreover, UNC5B silencing in the coculture setting demonstrated that UNC5B in the neighboring ECs is required for the barrier integrity enhanced by R-Ras of adjacent cells (Figure 4H). Downregulation of UNC5B and impaired barrier integrity (judged by reduced VE-cadherin staining) were observed in the neonatal retinal endothelium of *Rras*^*ΔEC*^ mice, corroborating the *in vitro* results (Figure 4I). To further demonstrate the importance of R-Ras for the barrier integrity of neighboring ECs, we prepared coculture monolayers composed of parental ECs with EC^mock^ or EC^RRas38V^ at a 3:1 ratio in Transwell inserts and examined dextran leakage. The coculture with EC^RRas38V^ limited the permeability of the EC monolayer; however, this effect was nullified by silencing Jagged1 in EC^RRas38V^ (Figure 5A).

In consonance with these *in vitro* observations, lung capillaries of *Rras*^*ΔEC*^ mice showed reduced Unc5b accumulation and increased extravascular fibrinogen, indicative of increased vessel leakiness (Figure 5B). Vascular permeability in the adult retinas was evaluated by perfusion of an amine-reactive ester derivative of biotin (sulfo-NHS-biotin). The perfused sulfo-NHS-biotin remained within blood vessels in control mice, confirming the intact BRB. In contrast, sulfo-NHS-biotin was detected in extravascular spaces in perfused *Rras*^*ΔEC*^ mice, indicating BRB breakdown in these animals (Figure 5C), an observation consistent with reduced VE-cadherin and UNC5B expression in the retina of these mice (Figure 4I). Corroborating this result, the accumulation of extravascular fibrinogen was detected in the retinas of *Rras*^*ΔEC*^ mice (Figure S8A). Furthermore, the *Rras*^*ΔEC*^ hippocampus exhibited significantly increased extravascular fibrinogen accumulation, indicating blood-brain barrier (BBB) disruption (Figure 5D). The BBB breakdown was accompanied by a significant decrease in Hey1 and Unc5b in these ECs (Figure 5E and S8B). These results support the idea that R-Ras signaling strengthens the barrier function of neighboring ECs through the Jagged1-Notch intercellular signaling and that UNC5B is a key effector downstream of this crosstalk.

### Akt3 mediates Jagged1 upregulation by R-Ras

**Figure 6.**
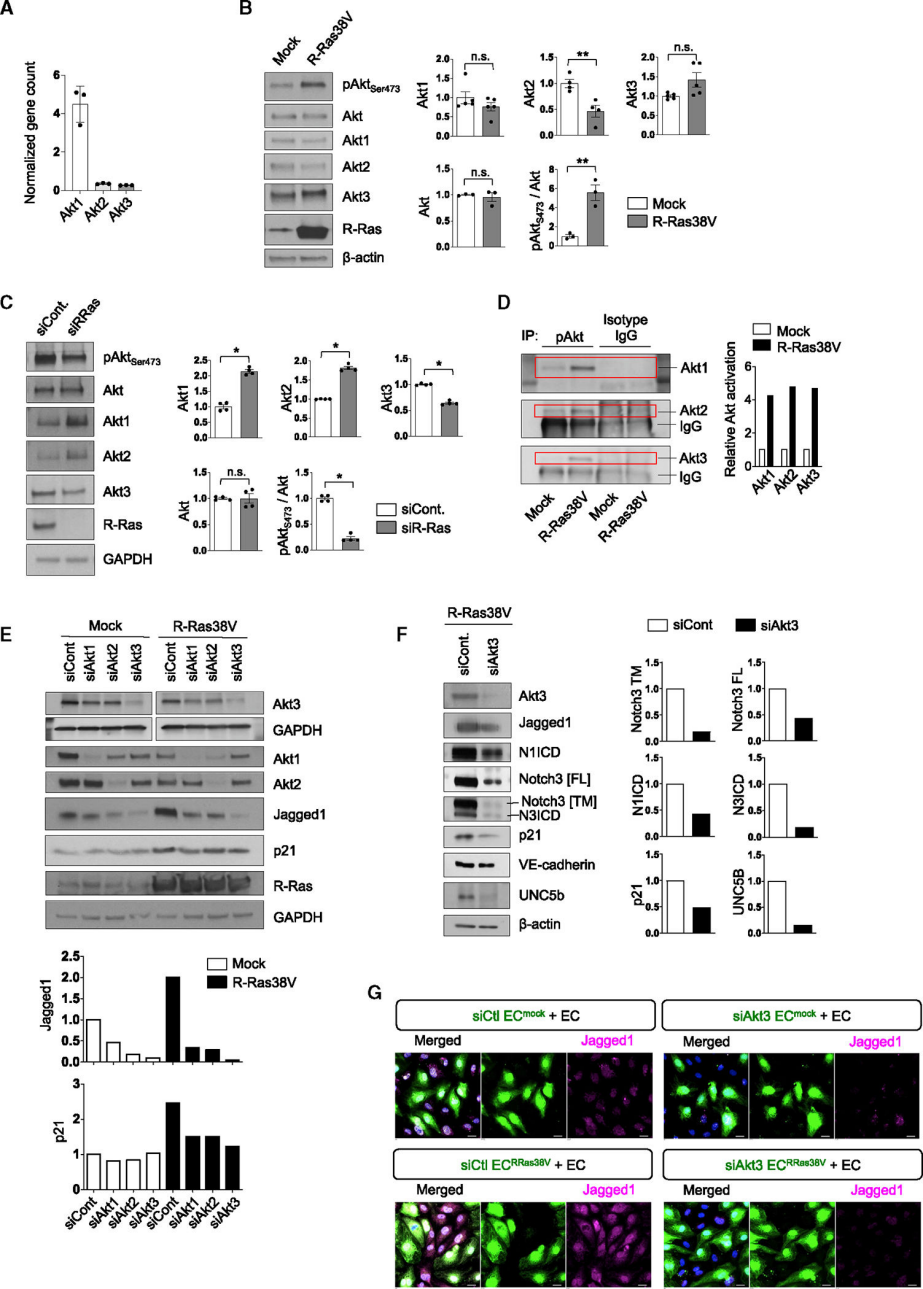
Jagged1 upregulation by R-Ras is Akt dependent (A) The gene expression levels in HUVECs were compared between Akt isoforms by RNA-seq. N = 3. Data are represented as the mean ± SD. (B and C) The protein level of each Akt isoform (N = 3–5) and Ser473 phosphorylation of all Akts were determined in R-Ras38V-transduced (B) or R-Ras-silenced (C) ECs by western blot. For quantification, pAkt_Ser473_ was normalized to the total Akt. Data are represented as the mean ± SEM. (D) Phosphorylation of each Akt isoform was determined in mock- or R-Ras38V-transduced ECs by immunoprecipitation of the total pAkt_Ser473_ followed by western blot of each isoform. (E) Each Akt isoform was silenced in mock- or R-Ras38V-transduced ECs and the protein levels of Jagged1 and p21 were determined in these cells. (F) The Akt3 isoform was silenced in R-Ras38V-transduced ECs, and Notch1 and Notch3 activation as well as the protein levels of Jagged1, p21, VE-cadherin, and UNC5b were determined. Data are represented as the mean ± SEM. (G) Akt3 was silenced in mock- (EC^mock^) or R-Ras38V-transduced ECs (EC^RRas38V^) that were cocultured with parental ECs (EC), and Jagged1 expression was evaluated. *p < 0.05, **p < 0.01; n.s., not significant.

We next investigated the mediator of R-Ras signaling to upregulate Jagged1 in ECs. R-Ras activates PI3K-Akt signaling.^22^ Akt1 and Akt2 are known to upregulate Jagged1 in ECs, which leads to the stabilization of vascular smooth muscle cells through Notch3 activation.^10^ We, therefore, examined the three Akt isoforms as candidate effectors for R-Ras. An RNA-sequencing (RNA-seq) analysis of HUVECs cultured in growth medium showed that all *AKT* isoform genes are expressed in these cells, with *AKT1* expression being 6- to 12-fold higher than that of the other two isoforms (Figure 6A). R-Ras38V expression decreased the protein level of Akt2 by 50%, while Akt1 or Akt3 showed a tendency to decrease or increase, respectively; however, these changes were statistically insignificant (Figure 6B). On the other hand, R-Ras silencing increased Akt1 and Akt2 approximately 2-fold while decreasing Akt3 by ~40% (Figure 6C). The total Akt protein level was not affected by either R-Ras38V expression or R-Ras silencing (Figures 6B and 6C). The total Akt Ser473 phosphorylation was increased >6-fold by R-Ras38V and decreased 75% by R-Ras silencing, demonstrating the importance of R-Ras for Akt activity in ECs (Figures 6B and 6C). The activation of each Akt isoform was analyzed by immunoprecipitation of phosphorylated Akt followed by western blot of individual isoforms. This study demonstrated that R-Ras38V activates all three Akt isoforms (Figure 6D).

We next analyzed the relative contribution of each Akt isoform to Jagged1 expression. Silencing of each isoform significantly decreased Jagged1 (Figure 6E). Unexpectedly, despite the low expression level of Akt3 in ECs, the silencing of Akt3 had the strongest impact, nearly abolishing Jagged1 expression in both mock- and R-Ras38V-transduced cells (Figure 6E). As demonstrated above, R-Ras activates Notch1 and Notch3 (Figures 1A–1C), and p21 and UNC5B are upregulated by R-Ras in a Notch-dependent manner (Figures 2A–2F, 4E, S2B, and S2C). However, all these effects of R-Ras were reversed by Akt3 silencing (Figures 6E and 6F) in a manner similar to Notch inhibition or Jagged1 silencing (Figures 1H, 1I, 2F, and 6F). We also found that Akt3 silencing reduces Akt1 and Akt2 expression in mock-transduced ECs, indicating that Akt3 influences the levels of other Akt isoforms (Figure 6E). Combined observations establish the R-Ras-Akt-Jagged1-Notch1/3 axis, in which Akt3 plays a significant role.

Interestingly, a coculture experiment revealed that R-Ras38V transduction in ECs upregulates Jagged1 in the non-transduced neighboring cells as well as in the transduced cells (Figure 6G). Furthermore, silencing Akt3 in the mock- or R-Ras38V-transduced ECs, which constituted only 25% of the total EC population in the culture, resulted in strong downregulation of Jagged1 in nearly all ECs (Figure 6G). These observations suggest that Jagged1 in an EC upregulates Jagged1 in neighboring ECs via Jagged1-Notch juxtracrine signaling, resulting in propagation of its effect in the surrounding area. Supporting this idea, neutralizing Jagged1 activity by a blocking antibody resulted in decreased Jagged1 levels and downregulation of the Notch target Hes1 without altering Notch1 expression (Figures S9A, and S9B). This effect was accompanied by reduced accumulation of VE-cadherin at adherens junctions (Figure S9B and S9C). Conversely, EC stimulation with immobilized human recombinant Jagged1, but not scrambled Jagged1, resulted in Notch-dependent upregulation of R-Ras, Akt3, Jagged1, and VE-cadherin (Figure S9D), consistent with the idea that this signaling axis propagates to adjacent ECs. EC stimulation with an active Jagged1 peptide fragment produced similar effects (Figure S9E).

### Akt3 activation by R-Ras in an EC promotes quiescence of neighboring ECs

**Figure 7.**
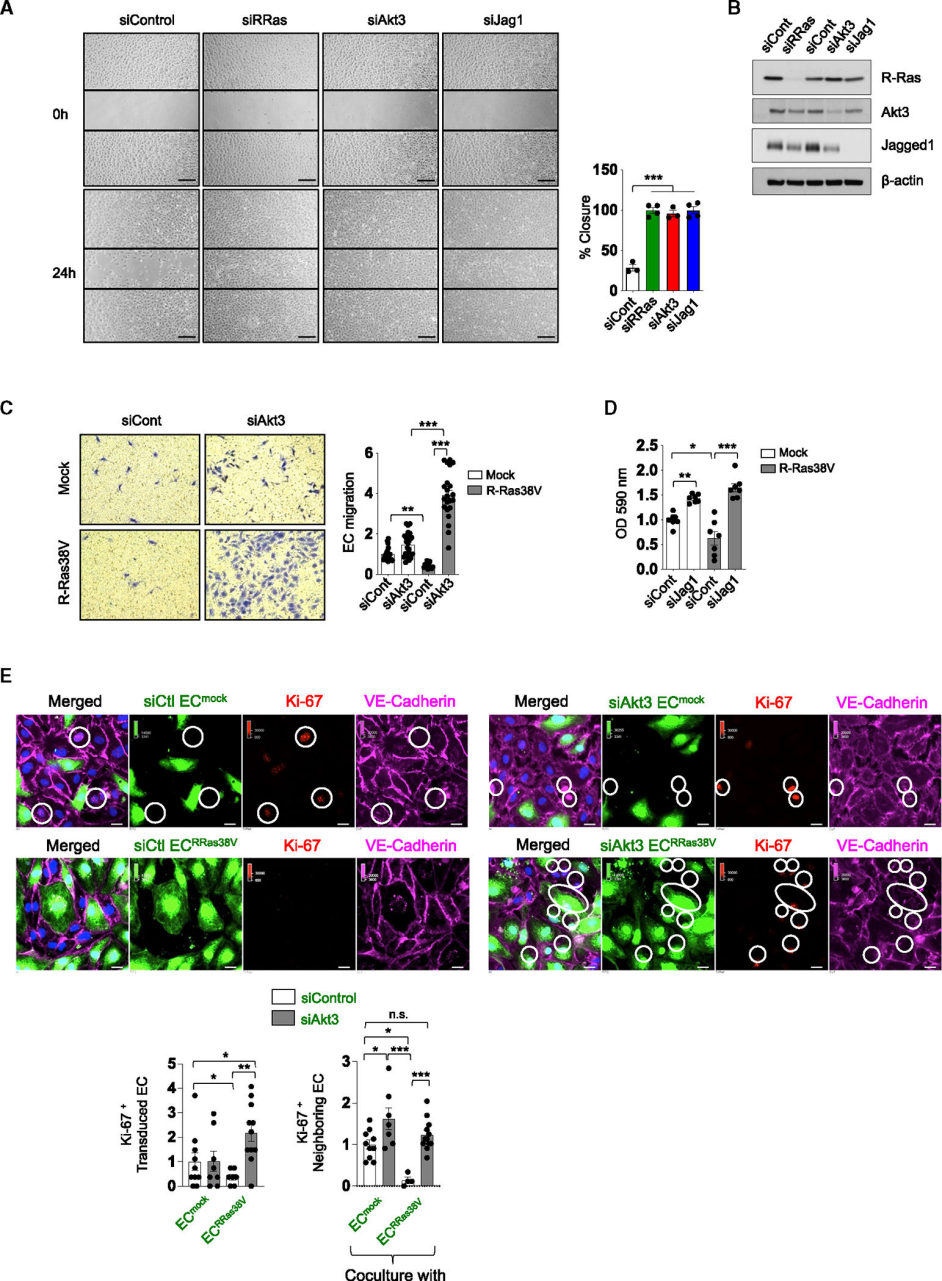
Akt3 inhibits EC migration and proliferation (A) Migration of R-Ras-, Akt3-, or Jagged1-silenced ECs was analyzed by a scratch-wound assay and quantified as the percentage of closure of the wound in 24 h. N = 3 culture dishes. Data are represented as the mean ± SEM. (B) si-knockdown efficacy on the scratch assay in (A) analyzed by western blot. (C) Akt3 was silenced in mock- or R-Ras38V-transduced ECs, and the migration of these cells was analyzed by Transwell migration assay. Cell migration is shown in relative values. N = 3 Transwell inserts, five or more pictures analyzed for each. Data are represented as the mean ± SEM. (D) Jagged1 was silenced in mock- or R-Ras38V-transduced ECs and an MTT assay was performed to analyze cell proliferation. N = 7 wells. Data are represented as the mean ± SEM. (E) EC^mock^ or EC^RRas38V^ with and without Akt3 silencing (green) were cocultured with unlabeled parental ECs at a 1:3 ratio. VE-cadherin and Ki-67 were stained and imaged 48 h later. Ki-67^+^ cells in the transduced EC population (green) and those in the parental (neighboring) EC population were quantified. Data are represented as the mean ± SEM in violin plots. *p < 0.05, **p < 0.01, ***p < 0.001; n.s., not significant. N = 4 wells, multiple pictures were analyzed for each well.

Little is known about the contributions of Akt3 to the regulation of angiogenic activities and endothelial barrier stability. We first analyzed the role of Akt3 in regulating EC migration. We found that Akt3 silencing increases EC migration (Figures 7A and 7B) similar to the effect of R-Ras or Jagged1 silencing (Figures 3F and 3G), demonstrating the inhibitory effects of Akt3. R-Ras38V inhibited EC migration (Figure 7C) as previously demonstrated^21^; however, R-Ras38V potentiated EC migration in the absence of Akt3 (Figures 7C and S10). R-Ras38V potentiated EC migration also in the absence of Notch (Figure 3H). These observations support the idea that Akt3 and Notch operate in the same R-Ras signaling axis.

We next examined the roles of Jagged1 and Akt3 in the regulation of EC proliferation downstream of R-Ras. MTT assays showed decreased EC proliferation by R-Ras38V; however, silencing of Jagged1 significantly increased proliferation in these cells as well as in the mock-transduced ECs expressing endogenous R-Ras (Figure 7D). The role of Akt3 was examined in a coculture setting. In this study, Akt3 was silenced in mock- (EC^mock^) or R-Ras38V-transduced ECs (EC^RRas38V^), and these ECs were cocultured with non-transduced ECs. Ki-67 staining demonstrated a 50% reduction in cycling ECs in the EC^RRas38V^ population, which was reversed by Akt3 silencing (Figure 7E). An over 2-fold increase in cycling cells was observed in Akt3-silenced EC^RRas38V^ cells compared with control EC^mock^ expressing Akt3, suggesting that R-Ras promotes EC proliferation in the absence of Akt3 (Figure 7E). Interestingly, coculturing with EC^RRas38V^ inhibited the cell cycle of non-transduced neighboring ECs by 90%, which was completely reversed by Akt3 silencing in EC^RRas38V^ (Figure 7E). The silencing of Akt3 in EC^mock^ also promoted the cell cycle of neighboring ECs by 50%, while it did not affect the cell cycle of EC^mock^ themselves (Figure 7E). Combined observations support the importance of Jagged1 and Akt3 downstream of R-Ras to suppress EC proliferation.

### R-Ras and Jagged1 are downregulated in hemangioma and kaposiform hemangioendothelioma

Vascular neoplasms like infantile hemangioma and hemangioendothelioma display common histopathologic features, such as mitotic ECs with gross abnormalities, including EC aggregates, anastomosis, and vacuolated cytosol. If left untreated, hemangiomas can grow extensively, causing serious medical complications.^44–46^ It has been reported that Jagged1 activation of Notch3 in pericytes resulted in a significant decrease in cell proliferation, inducing maturation of hemangioma pericytes by upregulation of p21.^47^ To determine whether endothelial R-Ras-Jagged1 axis might be compromised in the molecular pathophysiology of hemangioma in correlation with increased angiogenic activities, we performed immunofluorescence studies on clinical specimens of infantile hemangioma and kaposiform hemangioendothelioma (Figures S11 and S12A). Pediatric skin samples were used as a control. Immunostaining of these hemangiomas showed a strong downregulation of both R-Ras and Jagged1 compared with the endothelium of normal skin capillary vessels. The Notch target gene Hes1 was also strongly downregulated in hemangioma ECs (Figure S12B). These observations support the idea that endothelial Notch activation by the R-Ras-Jagged1 axis helps control EC proliferation.

## DISCUSSION

In this study, we demonstrated the importance of Jagged1-mediated intercellular signaling for the quiescence and barrier integrity of ECs. Jagged1 is upregulated by Akt upon activation by R-Ras. All three Akt isoforms influence Jagged1 expression in ECs, but Akt3 is the most prominent Akt isoform in this role, despite its low expression level compared with Akt1. Jagged1 then activates Notch to upregulate Hey1, Hes1, p21, p53, and Unc5b in adjacent cells. Thus, the R-Ras-Akt3-Jagged1-Notch pathway mediates a crosstalk between ECs, through which ECs enforce the quiescence and barrier stability of neighboring ECs. The observation of BBB breakdown in the hippocampal vasculature exhibiting downregulation of Hey1, Unc5b, and VE-cadherin upon R-Ras deficiency supports this idea.

Larrivée et al. previously reported that Unc5b is highly expressed in the embryonic and neonatal mouse brain, lung, and kidney vasculature but downregulated in the quiescent adult vasculature.^42^ In the current study, we showed that Unc5b is expressed at significant levels in both hippocampal and lung capillary endothelium of adult mice. Global *Unc5B* knockout is embryonically lethal in mice due to defects in blood vessels that lead to heart failure, whereas inducible EC-conditional knockout mice exhibit seizures and die as a result of BBB breakdown within 2 weeks of tamoxifen induction,^40^ demonstrating the importance of Unc5b for endothelial barrier function. We showed that the EC-specific loss of R-Ras diminishes Unc5b expression in the brain and lung capillary endothelium. Notably, the regulation of UNC5b itself depends on the close interaction between ECs, since the juxtacrine Notch activation by Jagged1 upregulates UNC5b in adjacent ECs. We also demonstrated that UNC5b is necessary to stabilize VE-cadherin at adherens junctions of ECs. Thus, this mode of intercellular signaling provides a positive feedback regulation mechanism for stable EC-EC interactions necessary for the endothelial barrier function. Disruption of this feedback loop results in the breakdown of the barrier, EC migration, and proliferation.

The results from our study represent a mechanism of contact inhibition enforced by R-Ras and transmitted by Jagged1 to the neighboring ECs. The disruption of Jagged1 impaired the barrier function between ECs and potentiated EC migration. Combining the current and previous findings,^21–23,27,28,30^ accumulating evidence suggests that R-Ras shifts angiogenesis from the vessel sprouting process to the maturation process. R-Ras is strongly downregulated in proliferating ECs but highly expressed in non-proliferative ECs of mature blood vessels.^21,22^ We propose that the vessel-stabilizing effect of R-Ras propagates from the mature portion of blood vessels to the growing sprouts via Jagged1-Notch as the new sprouts mature into stable blood vessels.

The ability of R-Ras to inhibit EC proliferation and migration explains the anti-angiogenic activities of this Ras protein. It is intriguing that R-Ras, which activates integrins and enhances EC adhesion to the ECM, inhibits EC proliferation and migration and exerts anti-angiogenic effects. Our study revealed that these inhibitory effects of R-Ras are Akt3 and Notch dependent. In the absence of Akt3 or Notch, R-Ras enhanced EC migration and cell-cycle progression. These observations suggest that the communications between neighboring ECs via the Akt3-Jagged1-Notch axis are central to the anti-angiogenic activities of R-Ras. Since silencing Akt3 alone has such strong effects, Akt3 may have a non-redundant role in EC regulations compared with other Akt isoforms. In addition to regulating endothelial stability, R-Ras is also important for the lumenization of capillary vessels^22^ and facilitates lymphocyte extravasation (diapedesis) through high endothelial venules in the lymph nodes.^48^ It is currently unknown whether Akt3 or Jagged1 is involved in these functions of R-Ras. The activation mechanism of R-Ras is incompletely understood, but several studies suggested various guanine nucleotide exchange factors, including Ras guanyl-releasing proteins, as direct activators of R-Ras.^31,49,50^ For instance, RasGRP2 activates R-Ras and, through it, Akt to inhibit EC apoptosis induced by Bax.^31^ Angiopoietin-1 and cAMP-elevating agents (e.g., forskolin) can temporarily elevate R-Ras activity in ECs^23,51^; however, a short-term activation of R-Ras may not be sufficient for the constant signaling required for the stability of the endothelium, for example, for the BBB. Further studies are warranted to investigate the mechanism of chronic activation of R-Ras.

Both Akt and Jagged1 are widely considered as angiogenesis stimulators. However, opposite EC-stabilizing activities have also been suggested. For instance, Akt activation by R-Ras stabilizes the microtubule cytoskeleton via GSK-3β inhibition, leading to endothelial lumenogenesis while inhibiting vessel sprouting and branching.^22^ The EC-specific inducible deletion of *Akt1* in established blood vessels exhibited increased capillary density in the heart of adult mice.^10^ Jagged1 is highly expressed in ECs of arterioles that have undergone vessel maturation in the postnatal retina.^5^ We also showed in this study that Jagged1 is abundant in ECs of normal adult vasculature; thus, the Jagged1 expression level does not necessarily correlate with angiogenic activities of ECs. On the other hand, Jagged1 presented to ECs by head and neck squamous cell carcinoma cells promotes tumor angiogenesis via Notch activation.^52^ Thus, significant evidence indicates that Jagged1 is involved in both angiogenesis activation and vascular quiescence. A possible explanation to reconcile the two opposing outcomes is that Jagged1 has differential effects depending on how it is presented to adjacent ECs. R-Ras has a profound effect on the actin and microtubule cytoskeletons and cell morphology^22,28^ as well as cell-cell interactions of ECs.^23^ It might be that, in the presence of these effects of R-Ras, Jagged1 is presented to Notch of neighboring ECs in a manner that generates angiostatic effects. In this regard, Jagged1 may be similar to angiopoietin-1 receptor Tie2, which has either angiogenic or angiostatic activity depending on its localization in the cell.^53^ Of note, Tie2 signaling at cell-cell junctions, which leads to an angiostatic effect, is thought to be mediated via Akt.^53^ In addition, angiopoietin-1 can activate R-Ras.^22^ The *pdgfb*-Cre-dependent EC-targeted deletion of *Jag1* impaired the vascularization of neonatal retina, the observation that established the pro-angiogenic role of Jagged1.^4^ However, in these mice, the loss of Jagged1 may have disrupted both EC growth at the angiogenic front and EC stability at the stem of the new sprouts, leading to poor survival of these ECs, resulting in overall low vascularization of the retina. The fact that R-Ras expression is low in proliferating ECs but high in quiescent ECs^21^ is consistent with the putative role of R-Ras in influencing the differential effects of Jagged1 on ECs.

Although the importance of Notch3 for vascular smooth muscle cell differentiation is well documented,^7,54,55^ little is known about the role of Notch3 in the regulation of ECs. Our findings suggest that Notch3 is an important player in the EC-EC interaction contributing to EC quiescence. We demonstrated that Notch3 is upregulated by R-Ras at the mRNA level, and the protein levels of Notch3 and N3ICD are increased accordingly. Notch3 and N3ICD levels are also regulated by Jagged1 in ECs, a finding similar to what was recently reported for EC-smooth muscle cell crosstalk, where the loss of endothelial *Jag1* downregulated Notch3 in smooth muscle cells in different vascular beds.^56^ Notch3 expression in vascular smooth muscle cells is autoregulated by Notch3 activation by endothelial Jagged1.^54^ Interestingly, we found that endothelial Notch3 (or Notch1) expression is regulated by Notch1 (or Notch3) activation by Jagged1 of neighboring ECs. Furthermore, R-Ras expression is regulated by Notch. These observations suggest that R-Ras in an EC upregulates R-Ras in adjacent ECs, resulting in propagation of the EC stabilizing effect to a broad area of the endothelium.

This study identified a previously unknown angiostatic activity of Akt3 and highlights the importance of Jagged1 as a downstream effector of this activity. The molecular pathway revealed in this study furthers our understanding of the key mechanism of endothelial quiescence and barrier stability. Mutations in the *JAG1* gene cause Alagille syndrome and associated vascular anomalies leading to intracranial hemorrhage and other serious complications.^14^ The loss of Jagged1-dependent EC stabilization may account for some of the conditions of this and other diseases involving blood vessel disruption.

### Limitations of the study

There are some elements of methodology that limit this research:

1. Retina whole-mount immunostaining as a technique to collect data has some limitations, such as the following:
  - Jagged1 and Notch1 are strongly expressed in many cell types in the retina: microglia, resident macrophages, pigment epithelium, photoreceptors (cones and rods), and other neurons, as well as pericytes and vascular smooth muscle cells. This makes it unfeasible to do proper evaluations and quantification of the expression of these proteins specifically in the endothelium by immunostaining.
  - Many antibodies do not work for whole-mount tissue staining for many reasons, for instance, because tissue-fixation methods and detergents used for cell permeabilization are incompatible with certain antibodies. Also, antigen retrieval treatment is inapplicable to whole-mount staining.
  - The expression of some of these proteins, including Jagged1, increases over time as the endothelium matures. Detection at early stages by whole-mount immunofluorescence is difficult.
2. It is not possible to use the wound-healing assay (scratch assay) to analyze cell migration to determine the effects of the silencing of *AKT1* or *AKT2* because these Akt isoforms are essential for cell growth, proliferation, survival, and cell health, and the silencing of either *AKT1* or *AKT2* does not allow the formation of confluent EC monolayers necessary for performing and properly assessing the assay. The use of Akt inhibitors causes a similar problem, reflecting the critical role of these kinases in EC health. This also applies to the coculture system to study juxtacrine signaling.

## STAR★METHODS

Detailed methods are provided in the online version of this paper and include the following:

### RESOURCE AVAILABILITY

#### Lead contact

Further information and requests for resources and reagents should be directed to and will be fulfilled by the lead contact, Masanobu Komatsu (mkomats1@jhmi.edu).

#### Materials availability

- This study did not generate new unique reagents.
- The *Rras*^*fl/fl*^ mouse line used in this study have been donated to the Jackson Laboratory: B6(SJL)-*Rras*^*tm1.1Masak*^/J. Strain#036202 [RRID:IMSR_JAX:036202]

#### Data and code availability

- RNA-seq data have been deposited at Mendeley and at GEO (NCBI) and are publicly available as of the date of publication. Accession numbers are listed in the key resources table. Original western blots have been deposited at Mendeley and are publicly available as of the date of publication. The DOI is listed in the key resources table.
- This paper does not report original code.
- Any additional information required to reanalyze the data reported in this paper is available from the lead contact upon request.

### EXPERIMENTAL MODEL AND STUDY PARTICIPANT DETAILS

#### Experimental animals

All procedures involving animals used in this study were approved by the Institutional Animal Care and Use Committee of Johns Hopkins University. *Rras*^fl/fl^ mice were generated by Ingenious targeting laboratory (Ronkonkoma, NY, USA)^30^. *Cdh5*-Cre mice were obtained from The Jackson Laboratory (B6;129-Tg(Cdh5-cre)1Spe/J). *Cdh5*-Cre mice were crossed with *Rras*^fl/fl^ mice to generate *Cdh5*-Cre^+^;*Rras*^fl/fl^ endothelial cell-specific knockout mice (*Rras*^*ΔEC*^) as well as Cre-negative littermate control *Rras*^fl/fl^ mice. A PCR primer set 5’-AGCATCTTGTCACTGCTGTATATAAGCCC-3’ and 5’-CTAGCCAGATTACCATTCCC-3’ was used to detect the *Rras*^*flox*^ allele. For genotyping of *Cdh5*-Cre, the following commercial primers were used: 5’-GCCTGCATTACCGGTCGATGCAACGA-3’ and 5’-GTGGCAGATGGCGCGGCAACACCATT-3’. All primers were purchased from IDT, Inc. (Illinois, USA), and GoTaq^®^ Green Master Mix (pre-mixed ready-to-use solution, M7122, Promega, WI, USA) was used for PCR reaction.

### METHOD DETAILS

#### Lentivirus transduction

Human umbilical vein endothelial cells (HUVEC) were used for *in vitro* studies. These ECs were transduced with a lentiviral vector carrying cDNA for a constitutively active form of R-Ras (hereafter, R-Ras38V), or an insertless vector (mock) for 18 hours. Media was then replaced with fresh media and the cells were cultured for three days at 37°C before the use for experiments.

#### RNA interference

HUVEC cells were transfected at approximately 70% confluency in complete medium (antibiotic-free) with 10nM siRNA targeting *RRAS*, *AKT1*, *AKT2*, *AKT3*, *JAG1, NOTCH1, NOTCH3* or *UNC5B* (ambion, Life Technologies, TX; see Table S1), using Lipofectamine RNAiMAX reagent (ThermoFisher Scientific). After 24 h, cells were washed with PBS and re-fed with fresh growth media and cultured for additional 48 hours.

#### Real-time quantitative PCR (RT-qPCR)

Total RNA was isolated from ECs using NucleoSpin^®^ RNA Plus (#740984.50, Takara Bio USA, CA). cDNA was synthetized by using Superscript IV Reverse Transcriptase^™^ (18090010, ThermoFisher Scientific, MA). Primer sequences used are listed in Table S2. Amplification and detection were performed using the Power SYBR Green PCR Master Mix (#4367659, ThermoFisher Scientific). The relative gene expression level was determined using the comparative *Ct* (Livak) method for triplicate reactions, normalizing to the expression level of 18S mRNA. The amplification efficiencies of targets and the reference genes were determined for each set of primers by using increasingly doubling concentrations of cDNA.

#### Endothelial cell co-culture

To examine the effect of endothelial R-Ras on the activation of Notch target genes in neighboring ECs, non-transduced ECs were co-cultured with fluorescently labeled mock- or R-Ras38V-transduced ECs. For this, mock- or R-Ras38V-tranduced ECs were incubated with 5 μM Cell Tracker ^™^ green (C7025, Invitrogen) for 45 minutes in red phenol-free basal medium (CC3129, Lonza). Cells were then washed with PBS and incubated in complete growth media overnight. Next day, the non-transduced, unlabeled ECs and the green-fluorescent transduced ECs were washed with PBS, trypsinized, and seeded together onto a new culture plate at 3:1 ratio, and co-cultured for 48h. Then, the cells were washed, fixed in 4% paraformaldehyde, permeabilized with 0.1% Triton X-100, blocked in 1% BSA-PBS solution and incubated with primary antibodies (see Key Resources Table) for immunostaining studies.

#### BrdU incorporation assay

To identify proliferating cells, 5-bromo-2’-deoxyuridine (BrdU), an analog of the nucleoside thymidine was used following the manufacturer’s instructions (ab142567, abcam). R-Ras38V or mock-transduced cells were seeded onto 4-well chamber glass slides (#154526 Lab-Tek II, Thermo Fisher Scientific) at 40,000 cells/well and labeled with 10 μM BrdU labeling solution at 37°C for 24 hours in a CO_2_ incubator. Then, cells were washed in PBS, fixed and permeabilized with 0.05% Triton X-100. Then, cells were incubated with 2M HCl for DNA hydrolysis at 37°C for 20 minutes, followed by incubation with 0.1M Sodium borate pH 8.5 for 30 minutes at room temperature for neutralization. As a negative control for BrdU detection, no hydrolysis was performed. Then, cells were thoroughly washed with PBS and blocked with 1% BSA-PBS solution for 1 hour, followed by incubation with anti-BrdU antibody in conjunction with anti-Ki-67 antibody to label proliferating cells. Alexa-conjugated secondary antibodies were used to detect the primary antibodies and DAPI was used to label nuclei.

#### MTT assay

Proliferation of R-Ras38V or mock-transduced HUVEC was measured using the MTT assay kit (ab211091, Abcam, Cambridge, MA, USA) following the manufacturer’s instructions. Transduced ECs were seeded in 24-well plate at 20,000 cells/well. The next day, the culture medium was replaced with 50 μL of serum-free phenol red-free EGM-2 basal medium plus 50 μL of MTT working solution. The culture was incubated at 37°C for 3 h and then supplemented with 150 μL of MTT solvent. After incubation on a shaker at room temperature for 15 min, the absorption at 590 nm was recorded using the EnVision 2105 Plate Reader (Perkin Elmer, MA, USA).

#### Scratch-wound assay

To measure EC migration, early passage mock- or R-Ras38V-transduced HUVEC cells were grown to confluence in 60 mm dish, and a narrow wound was inflicted by scratching the dish surface with a pipette tip. The scratch wound was imaged immediately after the scratch and after 12 and 24 h by bright field microscopy (Nikon Eclipse TS100) and NIS-Elements 5.20.01 software. To study the effect of R-Ras, Akt3 or Jagged1 on EC migration, cells were transfected at approximately 80% confluency in complete medium (antibiotic-free) with 10 nM siRNA targeting *RRAS*, *AKT3*, or *JAG1* (ambion, Life Technologies, TX) using Lipofectamine RNAiMax (ThermoFisher Scientific). Cells were replaced with fresh growth medium 24 h later, and the scratch was inflicted to the confluent EC monolayer after additional 24 hrs. The area of the wound closure was measured using ImageJ software.

#### Transwell-migration assay

ECs were seeded in 6.5 mm Transwell culture inserts with 8.0 μm pore size (#3422, Corning) at a density of 40,000 cells per insert and incubated with complete media in both top and lower chambers for 24 hrs. To analyze chemotactic cell migration, both chambers were washed with PBS, the lower chamber was filled with basal medium (EBM-2) supplemented with 10% FBS as a chemoattractant. The culture inserts were filled with basal medium. After 24 h, cells were washed twice with PBS, fixed for 2 minutes using 3.7% formaldehyde, permeabilized with 20% methanol for 20 minutes at room temperature and stained with 0.5% crystal violet solution in 20% methanol for 15 minutes. Culture inserts were then rinsed twice in PBS and non-migrated cells were scraped off from the inner/top surface of the inserts. Migrated cells on the bottom surface were imaged using an optical microscope (Nikon Eclipse TS100) and NIS-Elements 5.20.01 software and quantified using ImageJ software. To examine cell migration after gene silencing, cells were transfected with siRNAs for 24 h after seeding in the inserts. Culture medium was replaced 24h later in both chambers, and the chemoattractant was added after additional 24 hours.

#### Endothelial permeability assay

Jagged1-silenced Mock or R-Ras38V-transduced HUVEC cells were plated with non-transduced HUVEC cells ([1:2] ratio) into a 24-well Transwell chamber (total 20,000 cells/well, 0.4 μm pore size; Corning, NY, USA). Cells were cultured in EGM-2 (Lonza) to produce an EC monolayer. 48 hours later, cell culture medium was replaced by red-phenol free cell medium (EBM-2) in both lower and upper chambers, and 1 mg/mL FITC-labeled 70 kDa dextran (Millipore-Sigma) was added to the upper chamber. The fluorescence intensity was measured in the lower chamber at different time points using a fluorophotometer (EnVision 2105, PerkinElmer). An empty chamber with no cells was used as readout of 100% permeability.

#### Notch activation by recombinant Jagged1

The recombinant human Jagged1 Fc chimera (rhJagged1) contains the signal peptide and extracellular domain of JAG1 fused at the C terminus to the Fc portion of human IgG. To study the effect of Notch activation by Jagged1, 3.5 cm plates were coated with 5 μg/ml rhJagged1 (#1277-JG, R&D Systems, MN) for 2 hours at 37°C. The control plates were coated with 5 μg/ml BSA (Biolabs, New England) or 5 μg/ml scrambled Jagged1 (a scrambled sequence of JAG1; #RP20525, GenScript, NJ). HUVECs (passage 1) were seeded to these plates at 2×10^5^ cells. To block Notch activation, cells were treated with 10 μM DAPT (N-[N-(3, 5-difluorophenacetyl)-l-alanyl]-s-phenylglycinet-butyl ester), a γ–secretase inhibitor that hampers Notch cleavage (#565770, EMD Millipore, MA). Cells were lysed 48 hours later, and protein was extracted and quantified for western blot analysis.

#### Notch activation by Jagged1 peptide

A Jagged1 peptide that functionally mimics the full-length Jagged1 was obtained from a commercial source (#JAG-1-pep-100, StemRD, CA). HUVECs (passage 1) were seeded at 2×10^5^ cells in 6 cm plates and treated for 48 hours with the Jagged1 peptide (1 μg/ml) to activate Notch^57^. Cells were then lysed for western blot analysis.

#### Jagged1 neutralization assay

HUVEC cells were seeded at 40,000 cells/well in 4-well chamber slides (#154526, Lab-Tek) and grown to confluency. Cells were then treated with 5 μg/ml human anti-Jagged-1 antibody (#AF1277-SP, R&D Systems) to block Notch activation by Jagged1. The Jagged1 antibody was added again 24 hours later to the culture media to keep Jagged1 neutralized. Twenty-four hours later, cells were washed 3 times in PBS and fixed for immunofluorescence studies of VE-cadherin.

#### Protein extraction and western blot analysis

Proteins were extracted using a RIPA buffer (R3792, Teknova, CA) containing a protease inhibitor cocktail (Complete 50X, Roche, France), a phosphatase inhibitor cocktail (PhosII and PhosIII, Sigma-Aldrich) and mixed with Laemmli buffer (BP-111R, Boston Bioproducts, MA) for SDS-PAGE with Tris-glycine-SDS running buffer. Protein concentration was determined using a bicinchoninic acid assay (23227, Pierce ^™^, MA). 20μg of total protein was subjected to gel electrophoresis in 4–15% acrylamide gel and electroblotted onto PVDF membrane (162–0177 Bio-Rad). Membranes were blocked in 5% bovine serum albumin (BSA, 12659 EMD Millipore, MA) in Tris-buffered saline (TBS) for 1 hr at room temperature and incubated overnight at 4°C with one of the antibodies listed in the Key Resources Table.

#### Immunoprecipitation

For Immunoprecipitation of phosphorylated Akt, mock- or R-Ras38V-transduced ECs were lysed on ice for 10 min in 1X cell lysis buffer (#9803, Cell Signaling) containing protease and phosphatase inhibitor cocktails. Cells were scraped off and sonicated on ice three times for 5 sec each. After centrifugation, protein concentration was estimated and 400 μg of total protein was incubated with anti-phospho-Akt Ser473 antibody (1:100 dilution) overnight at 4°C with rotation to allow immunocomplex formation, followed by incubation for 20 min at room temperature with 20 μl of pre-washed Protein A magnetic beads (#73778, Cell Signaling). Beads were washed five times and resuspended with 40 μl 2X Laemmli buffer (161–0737, Bio-Rad). After SDS-PAGE was performed, anti-Akt1, -Akt2 or -Akt3 western blotting was carried out for the detection of phosphorylated Akt isoforms. Lysate of 20 μg protein (5%) was directly used for western blot to indicate the protein input for the immunoprecipitation. Isotype control (Rabbit DA1E mAb IgG, #3900, Cell Signaling) was used to show specific binding of the primary antibody in the immunoprecipitation.

#### Retinal vascular permeability assay

*Sulfo-NHS-LC-biotin perfusion in retina.* 12-week-old mice were perfused with sulfo-NHS-LC-biotin (Thermo Fisher Scientific #21335) at 0.75 μg/g bodyweight. The heart was exposed using iris scissors and forceps. After making an incision to the right atrium, 10 ml of sulfo-NHS-LC-biotin dissolved in PBS-CMF was perfused via the left ventricle for 10 min at the injection rate of 1 ml/min by using a 26-gauge needle. At the end of 10 min perfusion, sodium phosphate-buffered 10% formalin solution pH 7.4 was injected into the left ventricle. The eyes were then removed, and retinas were processed as described below. Biotinylated amines in the proteins were detected by streptavidin staining followed by confocal microscopy.

#### Retina whole-mount staining

Retinas were processed for immunostaining as previously described^30^. Briefly, eyes were enucleated, fixed in 4% paraformaldehyde for 1 hour at room temperature (RT) and then washed in cold PBS. Retinas were dissected under a stereoscope microscope (Nikon SMZ1270) and fixed for an additional hour. After several washes with PBS, retinas were incubated in blocking buffer and permeabilized overnight at 4°C. After this, retinas were equilibrated using Pblec solution at RT and incubated overnight in Pblec solution at 4°C with desired primary antibodies (see Key Resources Table) and isolectin B4 (IB4, L2895; Sigma-Aldrich) to label the retinal endothelium. Finally, retinas were mounted on the slide and imaged using a confocal microscope (Nikon A1R; Nikon Instruments, Melville, NY) and analyzed with Nikon NIS-Elements AR Analysis 5.21 software.

#### Immunofluorescence

##### *In vitro* studies:

Cells were seeded onto 4-well chamber slides and grown to confluency for 48–72 h. Cells were then washed three times with PBS, fixed in 4% paraformaldehyde for 15 min, permeabilized with 0.1% Triton X-100 for 15 min, blocked in 1% BSA-PBS solution for 1 hour and incubated overnight with primary antibodies (Key Resources Table). Next day, cells were washed three times with PBS for 5 min and incubated with secondary antibodies (Alexa Fluor 488, 555 and 597, Invitrogen, Paisley, UK; Table 1) for 45 minutes in the dark, followed by washing three times with PBS and incubation with 4’,6-diaminidino-2-phenylindole, dihydrochloride (DAPI, Invitrogen) for 10 min. Finally, cells were washed with PBS, mounted and sealed with Cytoseal 60. Slides were analyzed using a Nikon Eclipse 90i fluorescence microscope (Nikon, Tokyo, Japan). Images were quantified using NIS-Elements AR 5.21.03 software.

##### *In vivo* studies:

12-week-old *Rras*^*ΔEC*^ mice and control mice were examined. Tissue processing of the mouse lung was performed as previously described^58^. Lungs were inflated with a 1:1 PBS-OCT compound mixture using a 23G needle before dissection, embedded in OCT compound, and frozen in liquid nitrogen. 10 μm-frozen sections were obtained using a cryostat and submitted to immunostaining.

##### Human studies:

Clinical specimens of hemangiomas and control skin samples were obtained from Nationwide Children’s Hospital, Columbus, Ohio through the Cooperative Human Tissue Network (CHTN). Paraffin-embedded sections were rinsed twice for five minutes in xylene. After deparaffinization, xylene was removed with 100% ethanol, followed by hydration in a series of graded alcohol until 1X PBS was used. Then, antigen retrieval was performed using 1X Tris-EDTA pH 8.5 Buffer (E1161, Sigma) in a decloaking chamber. After unmasking the antigenic epitope, slides were rinsed in warm PBS 5 times 5 minutes each. Tissue was blocked for 1 h using TBS-0.025% Triton X-100 1% BSA 0.3M Glycine. Samples were incubated overnight at 4C with primary antibodies diluted in TBS-0.025% Triton X-100 1% BSA. The following day, slides were washed 3 times with PBS and secondary antibodies were applied for 1 h at room temperature. Then, slides were incubated with DAPI for 10 min, washed 3 times in PBS and mounted.

#### Cell senescence

Senescence associated β-galactosidase staining (#9860, Cell Signaling Technology, MA) was performed to study cell senescence. Cells were seeded in 35 mm plates, grown to confluency and rinsed with PBS. β-Galactosidase staining was performed following the manufacturer’s protocol. Briefly, cells were fixed for 15 min at room temperature and rinsed twice with PBS. Then, cells were stained at 37°C overnight in a dry incubator. Next day, cells were imaged under a microscope and checked for the development of blue color. As a positive control for the staining, mock- or R-Ras38V-transduced HUVEC were treated with 400 μM H_2_O_2_ for 2 hrs in complete medium, washed in PBS and re-fed with fresh medium for 4 days. Cells were monitor daily for morphological changes.

### QUANTIFICATION AND STATISTICAL ANALYSIS

#### Statistics

Data were presented as mean ± SEM. To analyze the expression of Notch receptors, ligands, and target genes, both at the protein and mRNA levels, data were compared between groups and the *p* values were determined using Student’s *t* test, with Welch’s correction when heteroscedasticity was detected. For the quantitative presentation of the different proteolytic fragments of Notch detected by the same antibody, data were presented in stacked vertical bars. For analyses involving three or more groups, One-way ANOVA was performed followed by a *Post hoc* test to determine the statistical significance between pairs of experimental groups. Tukey *post hoc* test was used except for analysis involving the comparison of multiple group means with a single control group (for scratch wound assay), for which Dunnett’s *post hoc* test was used. To analyze the effects of R-Ras and Jagged1 on EC permeability *in vitro* (dextran leakage experiments) and for the MTT assay, 2-way ANOVA with Bonferroni correction was used. For all analyses, p < 0.05 was considered significant. All statistical analyses were carried out using GraphPad Prism version 5.0b for Mac (GraphPad Software, La Jolla, CA, USA).

## Supplementary Material

1

## Figures and Tables

**KEY RESOURCES TABLE:** 

REAGENT or RESOURCE	SOURCE	IDENTIFIER

Antibodies		
Rabbit monoclonal anti-phospho Akt XP (Ser473) (D9E)	Cell Signaling Technology	Cat #4060; RRID:AB_2315049
Mouse monoclonal anti-BrdU (Bu20a)	Cell Signaling Technology	Cat #5292; RRID:AB_10548898
Rat monoclonal anti-CD31	BD Pharmingen	Cat #550274
Rabbit polyclonal anti-CD31	abcam	ab28364; RRID:AB_726362
Rabbit monoclonal anti-ERG	abcam	ab196149
Sheep polyclonal anti-Human fibrinogen-FITC	Bio-Rad	4440-8004F; RRID:AB_961497
Mouse monoclonal anti-Hes1 (E-5)-Alexa 647	Santa Cruz	Sc-166410; RRID:AB_2117960
Rabbit polyclonal anti-Hey1	GeneTex	GTX118007; RRID:AB_11168085
Rabbit polyclonal anti-Jagged1	abcam	ab7771; RRID:AB_2280547
Mouse monoclonal anti-Jagged1 Alexa647 (E-12)	Santa Cruz	sc-390177; RRID:AB_2892141
Goat polyclonal anti-Human Jagged1	R&D Systems	AF1277
Rabbit monoclonal anti-Ki67 (SP6)	GeneTex	GTX16667; RRID:AB_422351
Rabbit monoclonal anti-Notch1 (EP1238Y)	abcam	ab52627; RRID:AB_881725
Rabbit polyclonal anti-activated Notch1 (cleaved, N1ICD)	abcam	ab8925; RRID:AB_306863
Rabbit monoclonal anti-Notch3	abcam	ab23426; RRID:AB_776841
Rabbit monoclonal anti-Notch3 (D11B8)	Cell Signaling Technology	Cat #5276; RRID:AB_10560515
Rabbit monoclonal anti-p21	Cell Signaling Technology	Cat #2947; RRID:AB_823586
Rabbit monoclonal anti-p53	Cell Signaling Technology	Cat #2527; RRID:AB_10695803
Mouse monoclonal anti-R-Ras M01 (2E12)	Abnova	H00006237-M01; RRID:AB_464256
Rabbit monoclonal anti-UNC5B (D9M7Z)	Cell Signaling Technology	Cat #13851; RRID:AB_2798330
Rat anti-Mouse CD144 (11D4.1)	BD Pharmingen	Cat #555289
Mouse monoclonal anti-Human VE-cadherin (F8)	Santa Cruz	sc-9989; RRID:AB_2077957
Goat polyclonal anti-rabbit Alexa Fluor-555	Thermo Fisher	A21429
Goat polyclonal anti-rabbit Alexa Fluor-647	Thermo Fisher	A21244
Goat polyclonal anti-mouse Alexa Fluor-647	Thermo Fisher	A21236
Goat polyclonal anti-rat Alexa Fluor-405	Thermo Fisher	A48261
Goat polyclonal anti-rat Alexa Fluor-488	Thermo Fisher	A11006
Goat polyclonal anti-rat Alexa Fluor-555	Thermo Fisher	A21434
Goat polyclonal anti-rat Alexa Fluor-647	Thermo Fisher	A21247
Mouse monoclonal anti-Akt1 (2H10)	Cell Signaling Technology	Cat #2967; RRID:AB_331160
Rabbit monoclonal anti-Akt2 (D6G4)	Cell Signaling Technology	Cat #3063; RRID:AB_2225186
Rabbit monoclonal anti-Akt3 (62A8)	Cell Signaling Technology	Cat #3788; RRID:AB_2242534
Rabbit monoclonal anti-phospho Akt XP (Ser473) (D9E)	Cell Signaling Technology	Cat #4060; RRID:AB_2315049
Mouse monoclonal anti-β-actin	Sigma-Aldrich	A2228; RRID:AB_476697
Rabbit monoclonal anti-c-Myc (D84C12)	Cell Signaling Technology	Cat #5605; RRID:AB_1903938
Rabbit polyclonal anti-CD31	abcam	ab28364; RRID:AB_726362
Rabbit polyclonal anti-DLL4	Cell Signaling Technology	Cat #2589; RRID:AB_2092960
Mouse monoclonal anti-GAPDH (6C5)	Santa Cruz	sc-32233; RRID:AB_627679
Rabbit monoclonal anti-Hes1 (D6P2U)	Cell Signaling Technology	Cat #11998
Rabbit polyclonal anti-Hey1	Proteintech	19929-1-AP; RRID:AB_10646438
Rabbit monoclonal anti-Jagged1 (28H8)	Cell Signaling Technology	Cat #2620; RRID:AB_10693295
Rabbit monoclonal anti-Jagged2 (C23D2)	Cell Signaling Technology	Cat #2210; RRID:AB_823553
Rabbit monoclonal anti-Notch1 XP (D1E11)	Cell Signaling Technology	Cat #3608; RRID:AB_2153354
Rabbit monoclonal anti-cleaved Notch1 (D3B8)	Cell Signaling Technology	Cat #4147; RRID:AB_2153348
Rabbit monoclonal anti-Notch2 XP (D76A6)	Cell Signaling Technology	Cat #5732; RRID:AB_10693319
Rabbit monoclonal anti-Notch3 (D11B8)	Cell Signaling Technology	Cat #5276; RRID:AB_10560515
Mouse monoclonal anti-Notch4 (A-12)	Santa Cruz	sc-393893;
Rabbit monoclonal anti-p16 INK4A (D3W8G)	Cell Signaling Technology	Cat #92803; RRID:AB_2750891
Rabbit monoclonal anti-p21	Cell Signaling Technology	Cat #2947; RRID:AB_823586
Rabbit monoclonal anti-p53	Cell Signaling Technology	Cat #2527; RRID:AB_10695803
Rabbit polyclonal anti-RRas	AnaSpec	A4862
Mouse monoclonal anti-UKHC (F-5)	Santa Cruz	sc-133184; RRID:AB_2132389
Rabbit monoclonal anti-UNC5B (D9M7Z)	Cell Signaling Technology	Cat #13851; RRID:AB_2798330
Mouse monoclonal anti-Human VE-cadherin	Santa Cruz	sc-9989; RRID:AB_2077957
Goat polyclonal anti-mouse IgG HRP-conjugated	Promega	W402B
Goat polyclonal anti-rabbit IgG HRP-conjugated	Promega	W401B

Bacterial and virus strains		

Insertless control pLenti6/V5 Lentivirus expression vector and R-Ras38V.	ThermoFisher Scientific	N/A

Biological samples		

Hemangioma and Control tissue	Cooperative Human Tissue Network through Nationwide Children’s Hospital (CHTN)	N/A

Chemicals, peptides, and recombinant proteins		

Jag-1 protein active peptide fragment	StemRD	JAG-1-pep-100
Jag-1, scrambled	Genscript	RP20525
Recombinant Human Jagged1 Fc Chimera	R&D Systems	1277-JG-050
Gamma secretase inhibitor DAPT	EMD Millipore	565770
CellTracker Green CMDFA	Invitrogen	C7025
Isolectin B4 from *Bandeiraea simplicifolia*	Sigma-Aldrich	L 2895
Streptavidin Alexa-647	Invitrogen	S32357
EZ-Link Sulfo-NHS-Biotin	Thermo Scientific	21217
BrdU (5-bromo-2’-deoxyuridine), Thymidine analog	abcam	Ab142567
Gamma secretase inhibitor LY411575	Selleck Chemicals	S2714
Lipofectamine RNAiMAX	Thermo Fisher Scientific	13778-150

Critical commercial assays		

RNA isolation NucleoSpin RNA Plus	Takara Bio USA	740984.05
MTT Assay for cell proliferation	abcam	Ab211091

Deposited data		

Full Western blots	This paper	Mendaley Data: https://doi.org/10.17632/57mv55r8ys.2
RNA-seq data	This paper	GEO Database: GSE254958

Experimental models: Cell lines		

Human umbilical vein cells	Lonza	C2519A

Experimental models: Organisms/strains		

B6;129-Tg(Cdh5-cre)1Spe/J	The Jackson Laboratory	017968
Rras flox/flox	Ingenious targeting laboratory, Ronkonkoma, NY	https://doi.org/10.1159/000514555

Oligonucleotides		

For siRNA oligonucleotides	Table S1	N/A
For qPCR Primers	Table S2	N/A

Software and algorithms		

NIS-Elements Advanced Research 5.21.03	Nikon	N/A
Image J 1.48v	NIH	http://imagej.nih.gov/ij
GraphPad Prism 5 for Mac OS X	N/A	N/A

## Highlights

- Quiescent endothelial cells guide quiescence of neighboring cells via cell contact
- This contact inhibition is mediated by Akt3 and applied by Jagged1
- Cell contact provides feedback reinforcement of the barrier in the neighborhood
- R-Ras-Akt3-Jagged1-mediated intercellular crosstalk provides angiostasis
