# Supplementary material for: Akt3 activation by R-Ras in an endothelial cell enforces quiescence and barrier stability of neighboring endothelial cells via Jagged1
Source: Cell Rep. Author manuscript; Available in PMC 2024 Apr 28. (PMC11056028; doi:10.1016/j.celrep.2024.113837)
Supplement: 1 [file NIHMS1980909-supplement-1.pdf]

Cell Reports, Volume *43*

Supplemental information

Akt3 activation by R-Ras in an endothelial cell enforces quiescence and barrier stability of neighboring endothelial cells via Jagged1

Jose Luis Herrera and Masanobu Komatsu

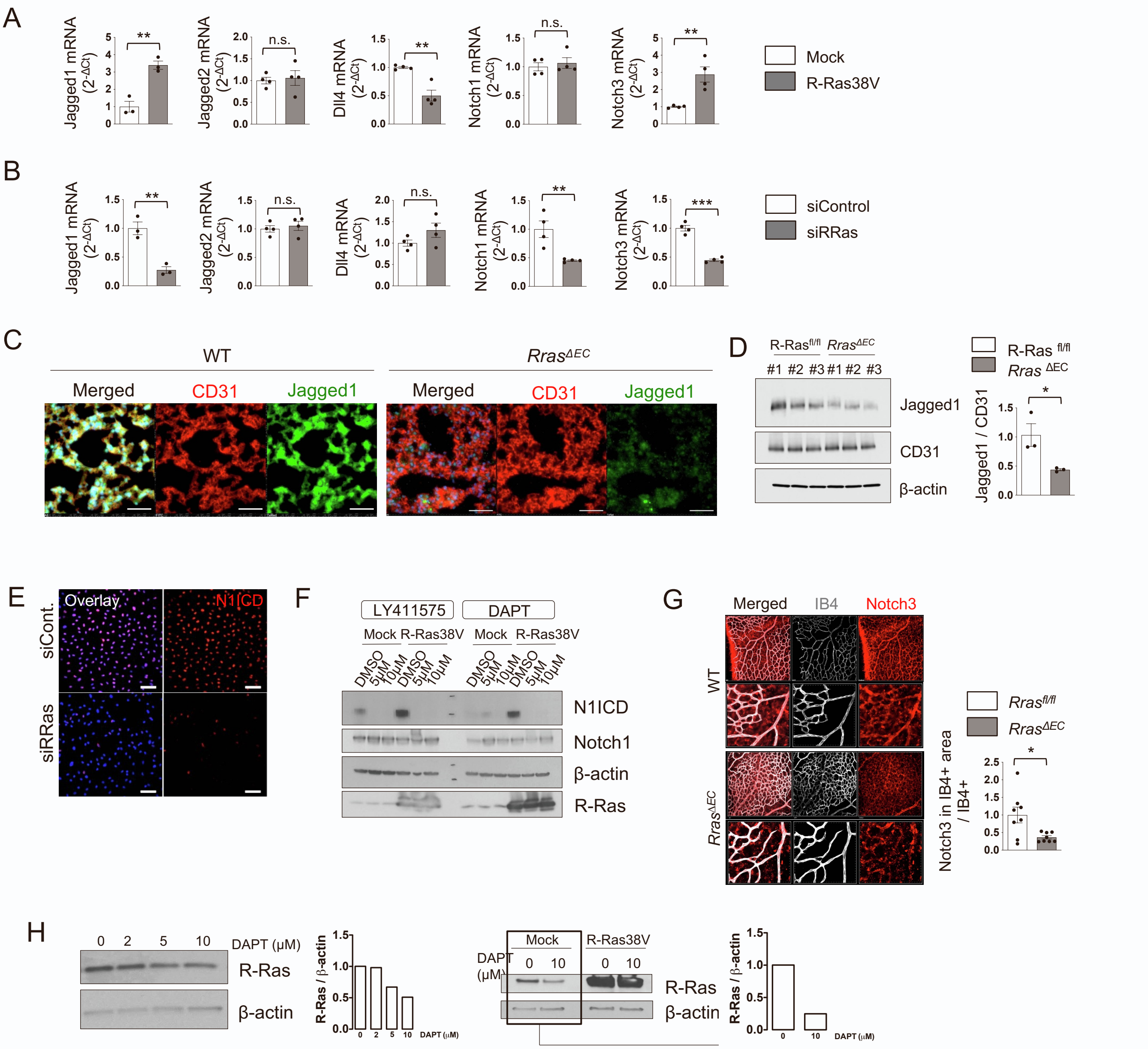

Supplementary Figure 1: R-Ras upregulates Jagged1 and activates Notch in ECs. Related to Figure 1.
a) mRNA analysis by RT-qPCR of various Notch ligands and receptors in mock or R-Ras38V-transduced ECs. N=3-4 b) mRNA analysis by RT-qPCR of various Notch ligands and receptors in and R-Ras-silenced ECs. N=3-4 c) Jagged1 expression in lung capillary ECs was analyzed in *cdh5*-Cre;$Rras^{f/f}$ mice ($Rras^{\Delta EC}$) and $Rras^{f/f}$ wild-type control mice (WT) by immunostaining. Scale bar, 50 μm d) Analysis of Jagged1 expression by western blot of whole lung tissue lysate from $Rras^{\Delta EC}$ mice. Three representative mice (#1-3) were shown for each group e) N1ICD nuclear accumulation was analyzed by immunofluorescence and DAPI staining of R-Ras-silenced ECs f) N1ICD level was analyzed by western blot in mock or R-Ras38V-transduced ECs after treatment for 48 hours with γ-secretase inhibitor LY411575 or DAPT at different doses g) Notch3 expression in postnatal retina ECs was analyzed in *cdh5*-Cre;$Rras^{f/f}$ mice ($Rras^{\Delta EC}$) and $Rras^{f/f}$ wild-type control mice (WT) by immunostaining. Notch3 intensity within CD31+ area was quantified and normalized to the total CD31+ area. N=3 mice. Data points indicate 2-3 pictures analyzed for each retina and are represented as mean ± SEM h) Notch inhibition decreases R-Ras expression in parental (top panel) or mock-transduced ECs in a DAPT dose dependent manner.

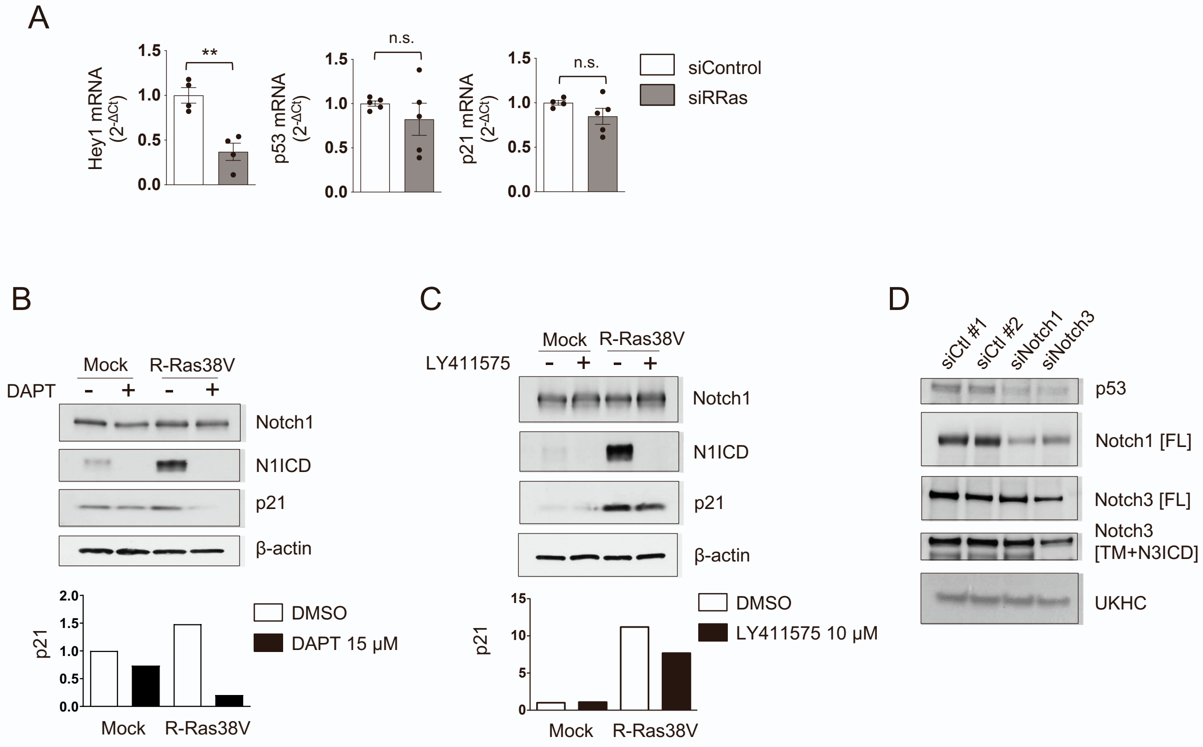

Supplementary Figure 2: R-Ras upregulates Hey1, p21, and p53 via Jagged1-Notch. Related to Figure 2.

a) mRNA analysis by RT-qPCR of Notch target genes in siControl or R-Ras-silenced ECs.

b) Analysis of p21 and N1ICD by western blot in mock or R-Ras38V-transduced ECs after treatment with 15 μM DAPT for 48 hours. The quantified band intensity was normalized to β-actin.

c) Analysis of p21 and N1ICD by western blot in mock or R-Ras38V-transduced ECs after treatment with 10 μM LY411575 for 48 hours.

d) Analysis of p53 by western blot in Notch1- or Notch3-silenced ECs. UKHC was used as a loading control.

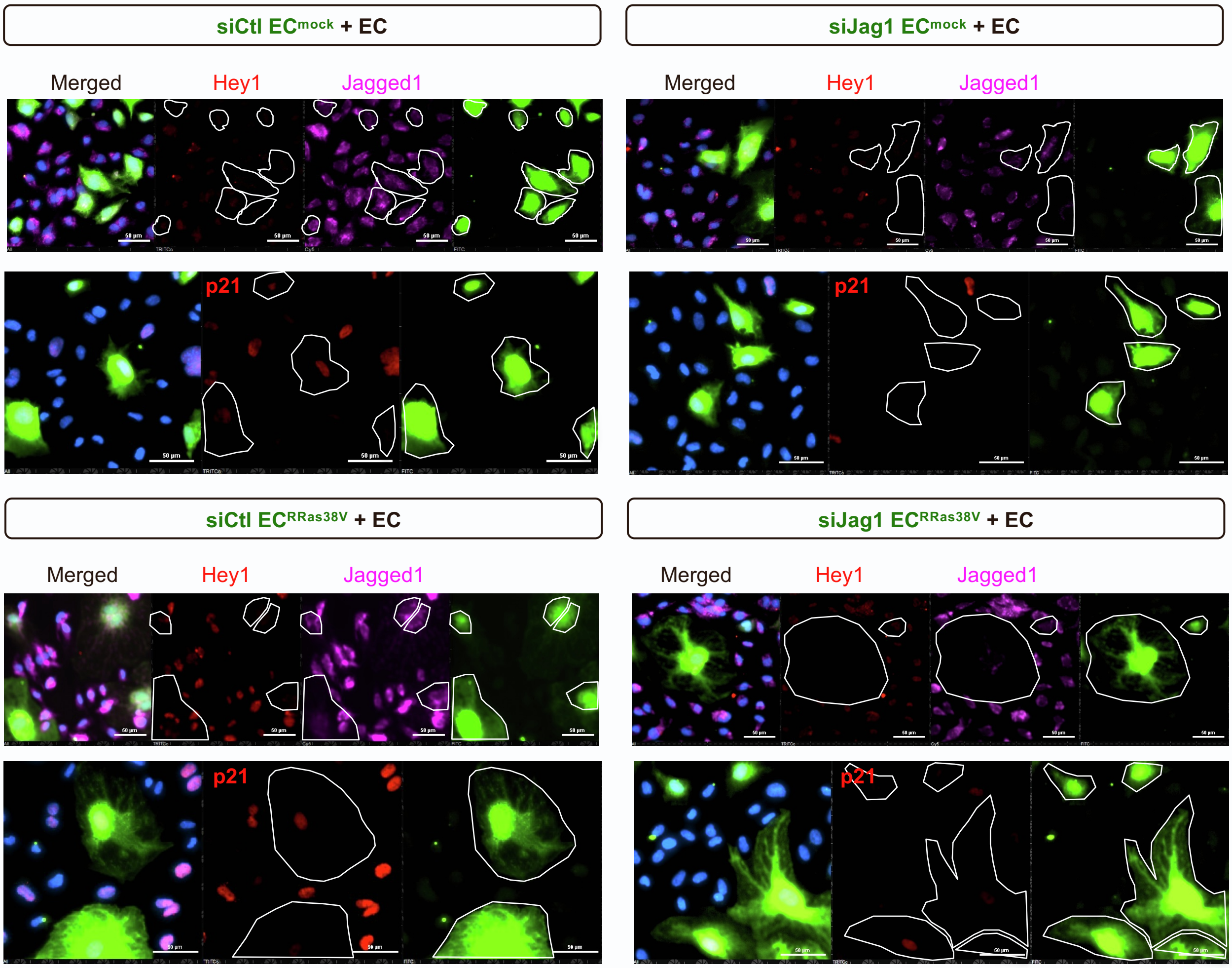

Supplementary Figure 3: Analysis of Notch-target genes in Jagged1- silenced mock or R-Ras38V-transduced ECs. Related to Figure 2G.

Jagged1 was silenced in mock control (EC^{mock}) or R-Ras38V-transduced ECs (EC^{RRas38V}) and fluorescently labeled (green), and then co-cultured with unlabeled, non-transduced parental ECs (EC) at 1:3 ratio. The cultures were immunostained with indicated antibodies.

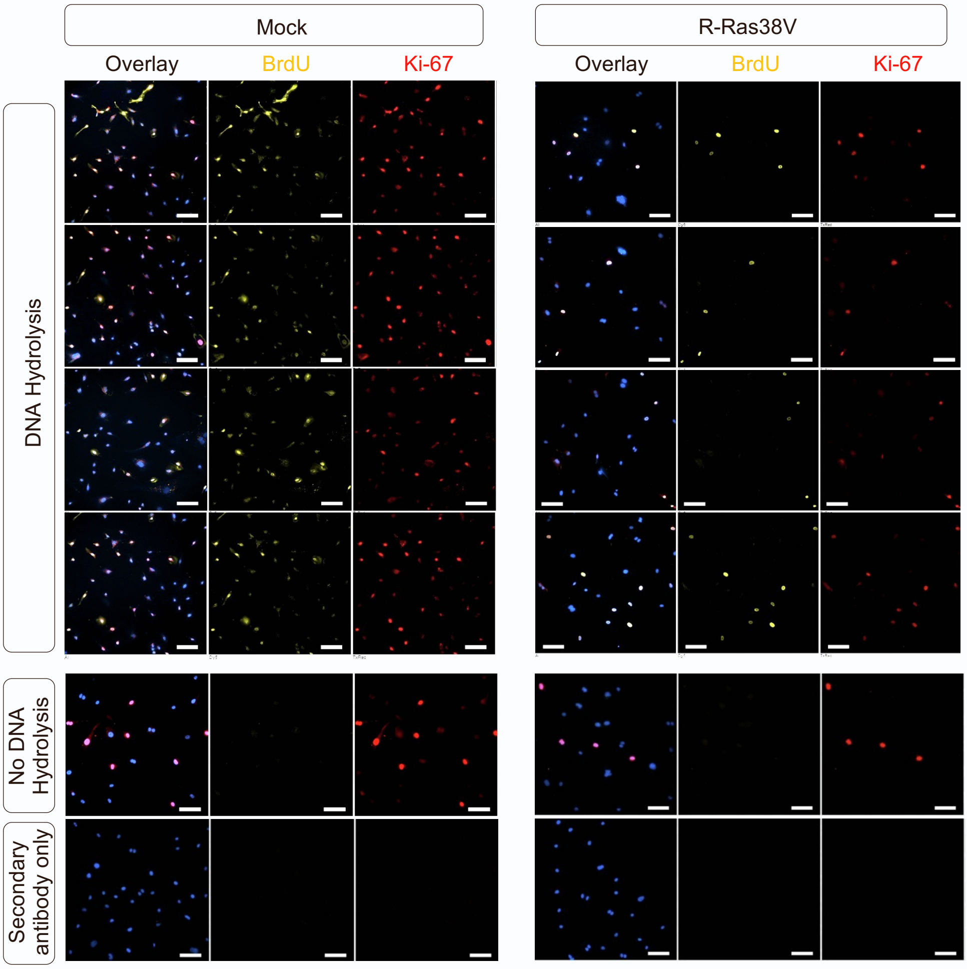

Supplementary Figure 4: BrdU incorporation assay to assess cell proliferation. Related to Figure 3B.

BrdU incorporation and Ki-67 staining of mock and R-Ras38V-expressing ECs to assess cell cycling.

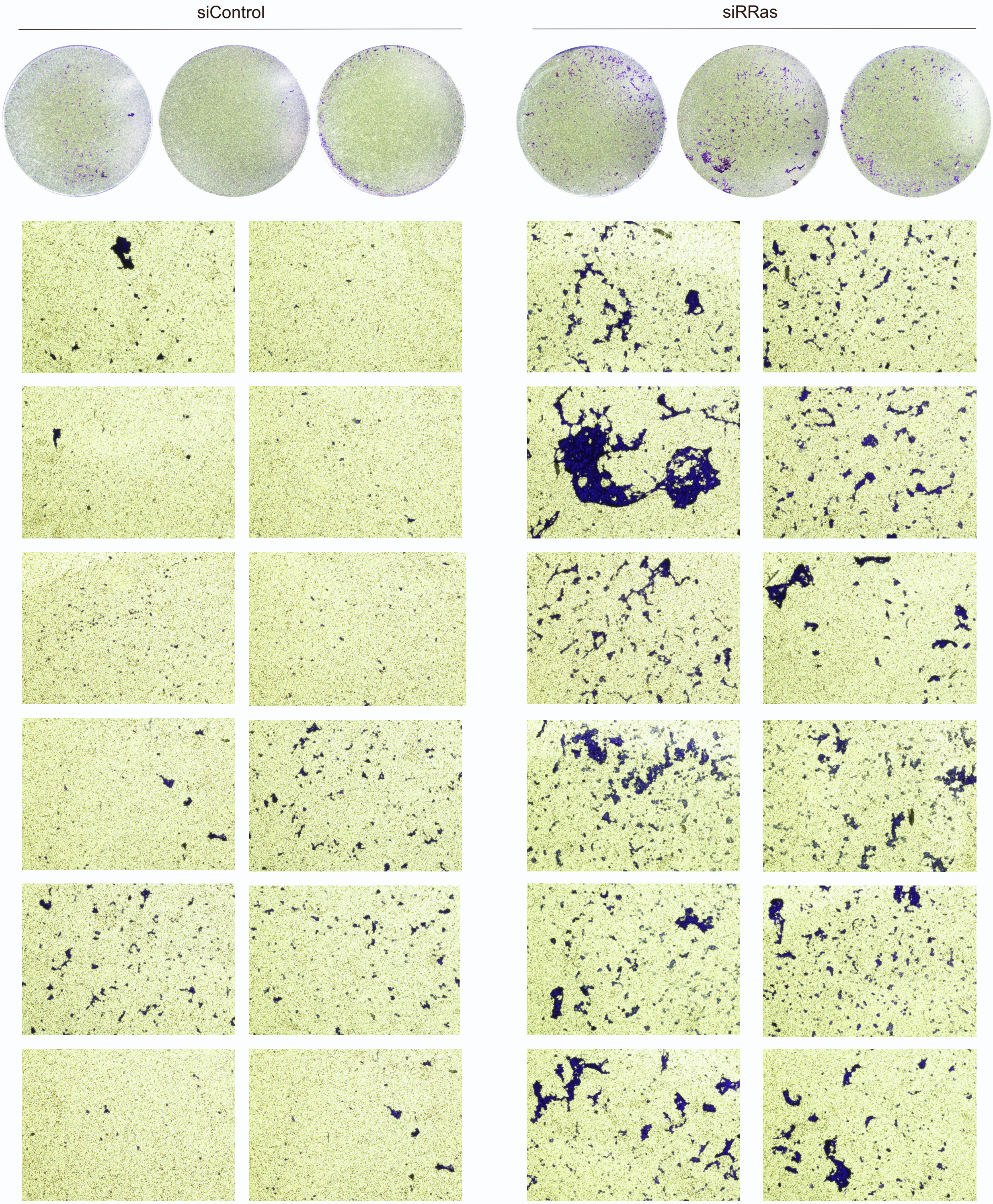

Supplementary Figure 5: Transwell migration assay in RRAS-silenced ECs. Related to Figure 3G.

Transwell migration assay of control and R-Ras-silenced ECs. Cells migrated to the bottom side of the Transwell inserts were stained with 0.5% crystal violet.

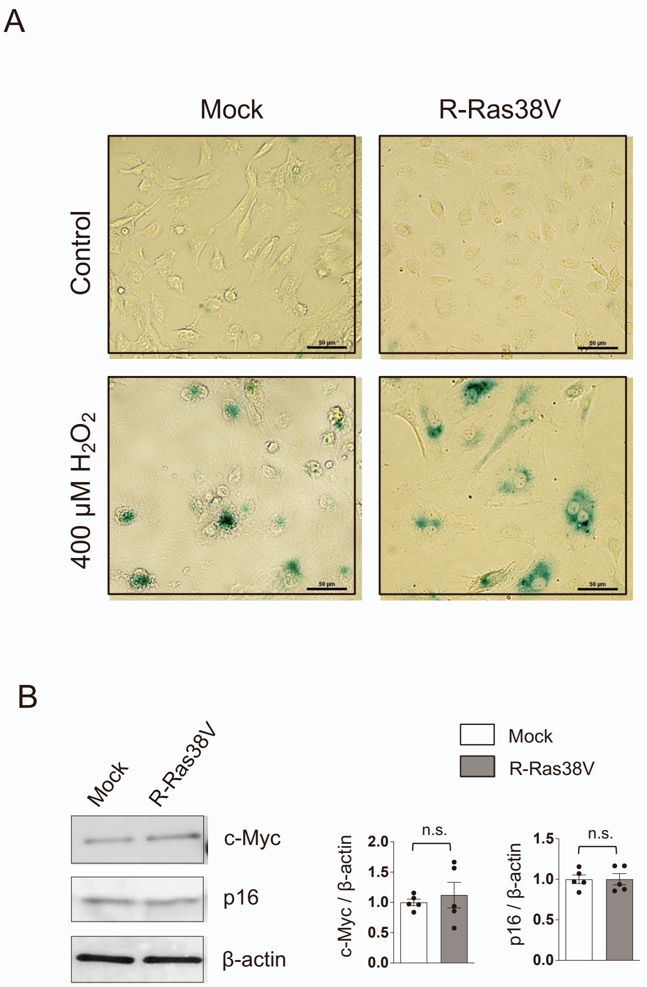

Supplementary Figure 6: Analysis of cell senescence in mock or R-Ras38V-transduced ECs.

a) Cell senescence was evaluated by β-Galactosidase staining in mock or R-Ras38V-transduced ECs. Hydrogen peroxide was used to induce cell senescence as a positive control.

b) Western blot analyses and quantification of the expression of senescence markers (c-Myc and p16) of mock or R-Ras-transduced ECs. N=5.

n.s., not significant

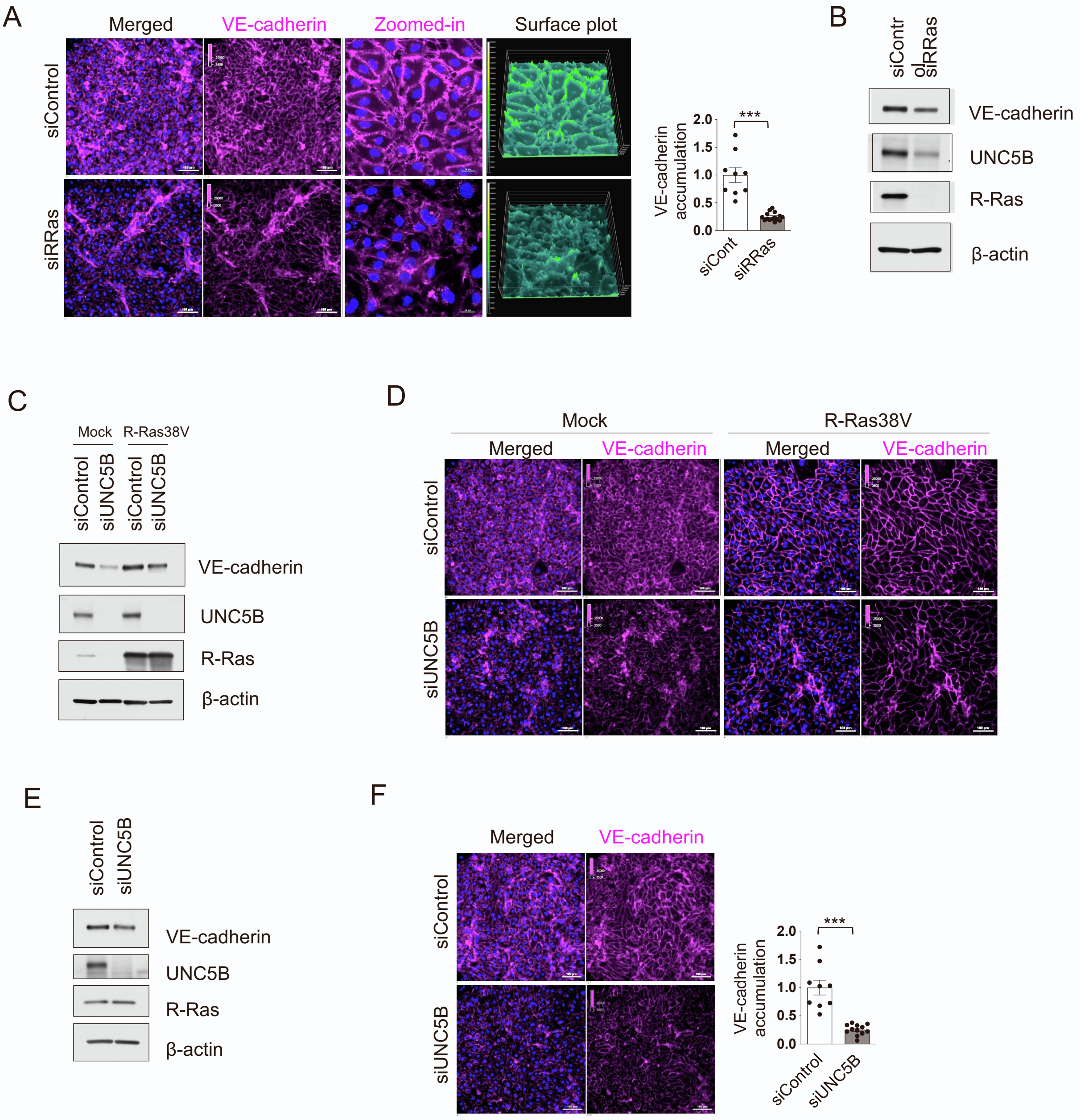

Supplementary Figure 7: Effect of R-Ras on barrier integrity and contribution of UNC5B. Related to Figure 4.

a) VE-cadherin accumulation at cell-cell junction (magenta) was visualized and quantified in control or R-Ras-silenced EC monolayer by immunofluorescence. DAPI nuclear staining (blue). Surface plots images indicate the levels of fluorescence intensity in color scale.

b) VE-cadherin and UNC5B expression were analyzed by western blot of control and R-Ras-silenced ECs.

c) The effect of UNC5B silencing on the VE-cadherin protein level was analyzed in mock or R-Ras38V-transduced ECs by western blot.

d) The effect of UNC5B silencing on VE-cadherin accumulation at the cell-cell junction was analyzed in monolayer of mock or R-Ras38V-transduced ECs by immunofluorescence.

e) VE-cadherin and UNC5B western blot of ECs with or without UNC5B silencing.

f) VE-cadherin immunofluorescence intensity of parental ECs with or without UNC5B silencing was quantified. N=4 wells, 2 or more pictures analyzed per well. ***p<0.001

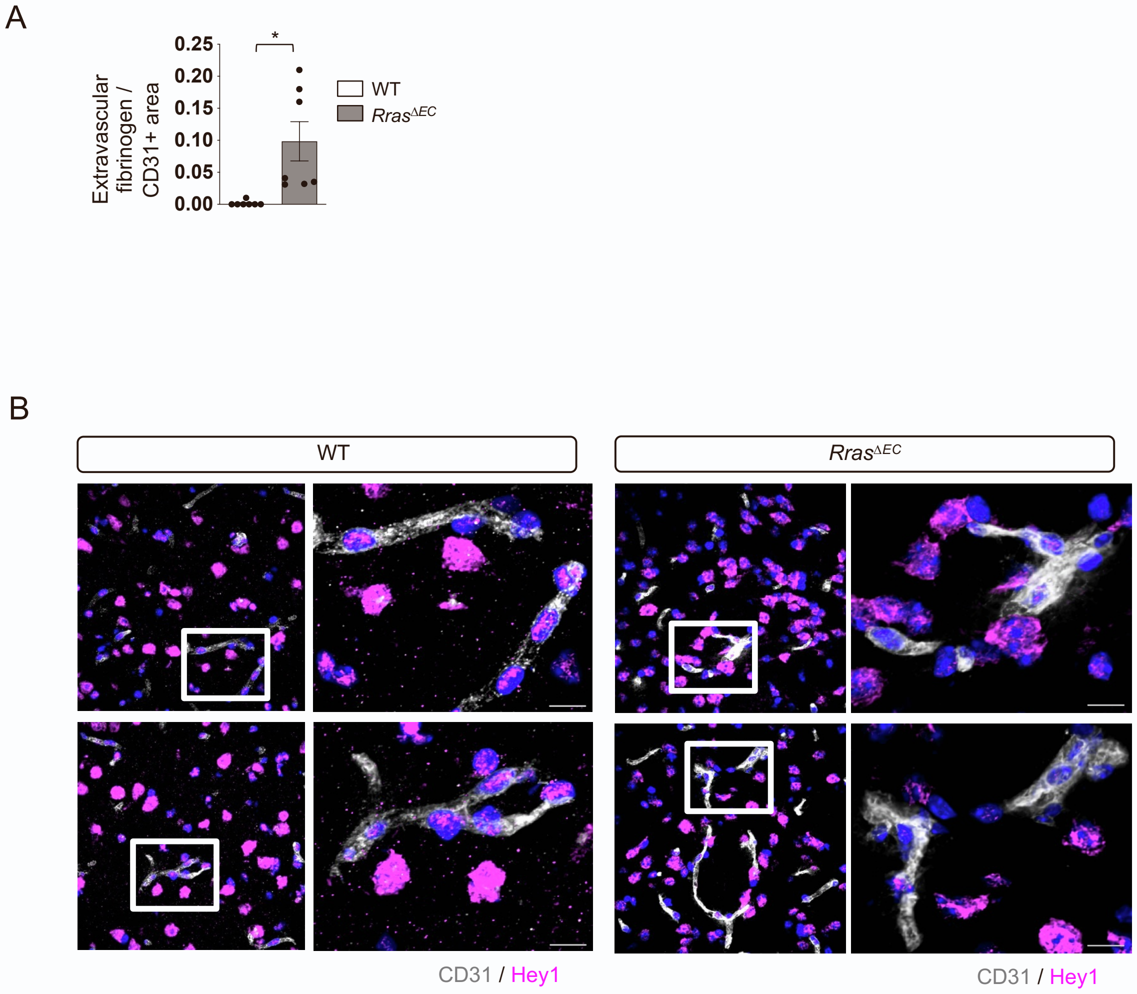

Supplementary Figure 8: Abnormalities in retinal and cerebral vasculature of *Rras*$^{\Delta EC}$ mice. Related to Figure 5.

a) Extravascular fibrinogen in the adult retina was analyzed by immunostaining in 3D-reconstructed confocal images. Fluorescence intensity (arbitrary unit) was normalized to the total CD31^{+} area. N = 7 retinas per group

b) CD31 and Hey1 immunofluorescence staining of the hippocampus. Hey1 (magenta) is readily detected in the nuclei (blue) of wild-type hippocampal microvascular ECs (white) consistent with the active Notch signaling. In comparison, a marked decrease of nuclear Hey1 is found in the *Rras*$^{\Delta EC}$ hippocampal microvascular ECs. Areas in white rectangles are magnified. Scale bars, 50 µm

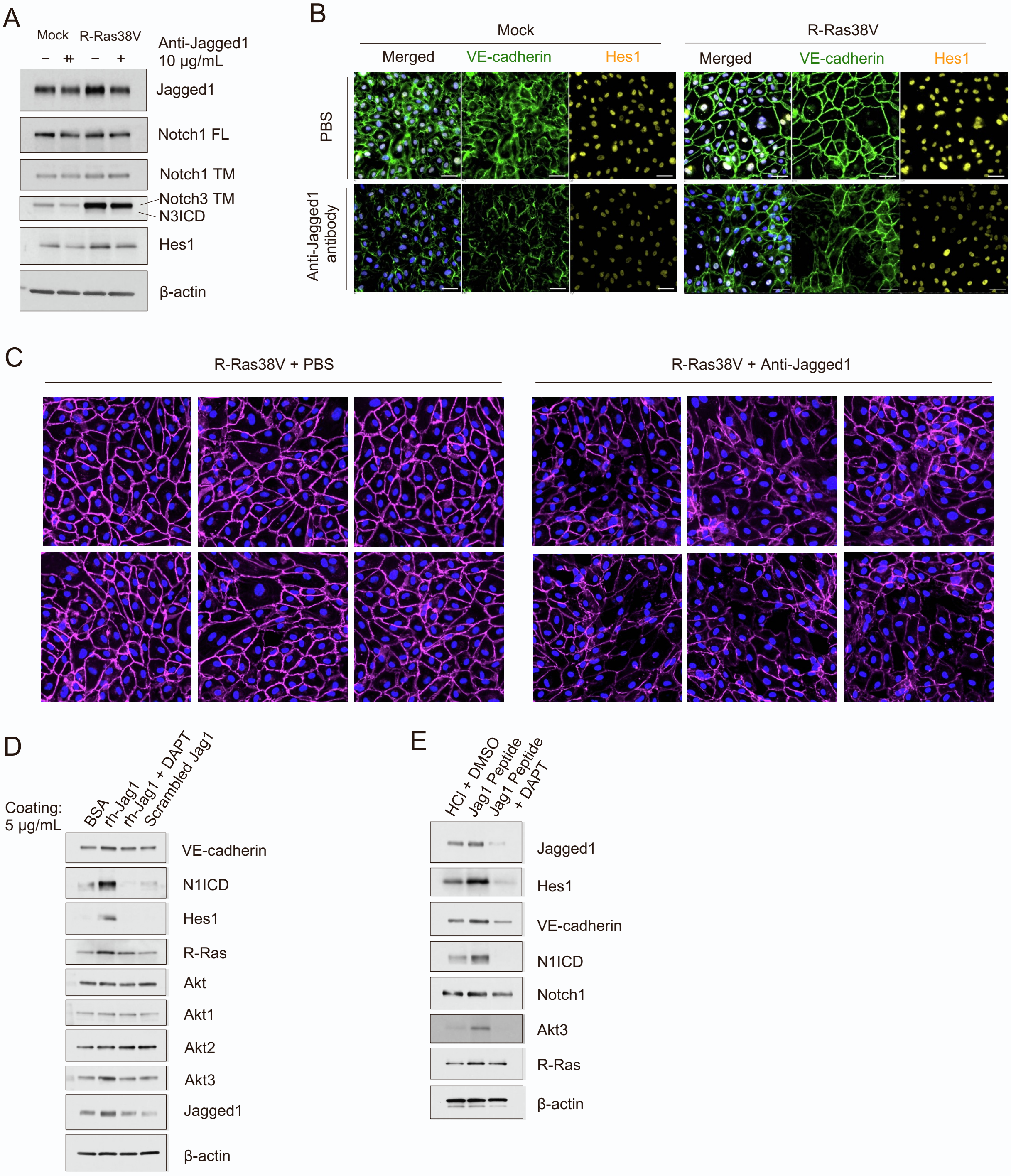

Supplementary Figure 9: Notch-dependent autoregulation of Jagged1.

a) Jagged-1 neutralization assay. ECs were exposed to anti-Jagged1 antibody (10 µg/ml) for 48 hours, and Jagged-1 expression was analyzed by western blot b) Effect of Jagged-1 neutralization on ECs. Mock or R-Ras38V-transduced ECs were exposed to anti-Jagged1 antibody for 48 hours, and VE-cadherin accumulation and Hes1 levels were analyzed in cell monolayers by immunofluorescence c) Effect of Jagged1 neutralization on VE-cadherin accumulation in mock or R-Ras38V-expressing ECs d) The effects of Notch activation by immobilized recombinant human Jagged1 (rh-Jag1). ECs were cultured on Jagged1-coated plates for 48 hours, and the expression of VE-cadherin, R-Ras, Akt isoforms, and Jagged-1 was determined by western blot. A scrambled Jagged1 was used as a negative control e) ECs were exposed to a Jagged1 peptide for 48 hours to activate Notch signaling, and the effects on VE-cadherin, R-Ras, Akt isoforms, and Jagged-1 expression were analyzed by western blot.

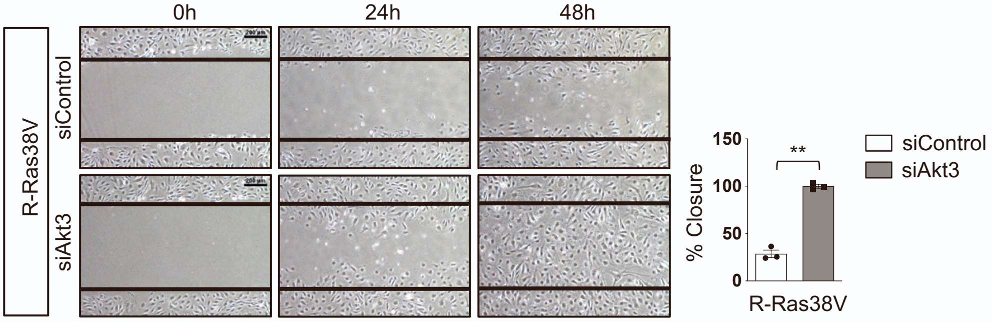

Supplementary Figure 10: Akt3 silencing reverses inhibition of EC migration by R-Ras. Related to Figure 6.

Scratch wound assay of R-Ras38V-transduced ECs upon silencing of Akt3. Data is presented as percentage of wound closure after 48 hours. N=3 culture dishes were analyzed.

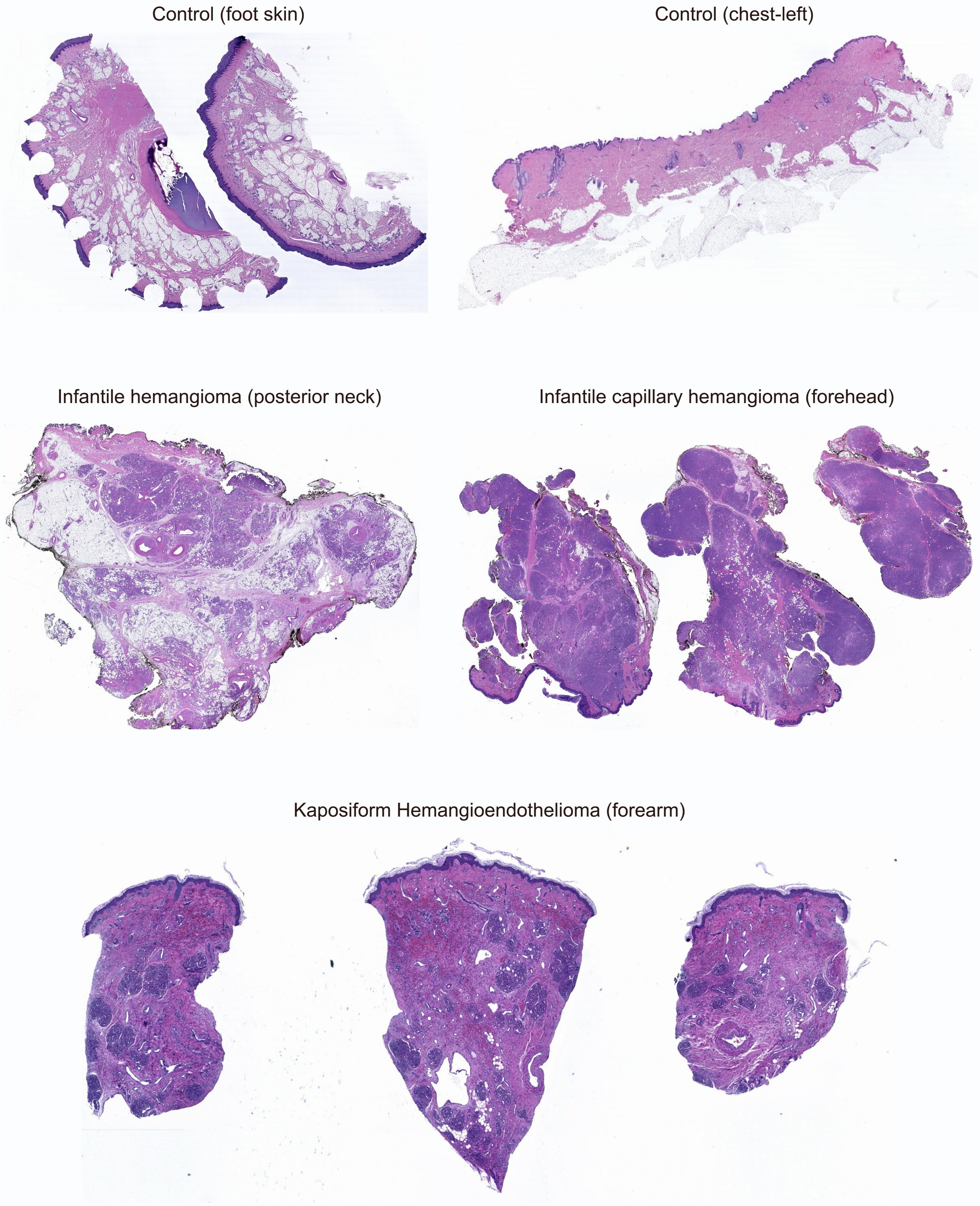

Supplementary Figure 11: Hematoxilin and Eosin staining of pediatric vascular tumors (hemangiomas). Standard hematoxylin and eosin staining (H&E) was used to confirm the histological diagnosis of several hemangioma specimens. Pediatric skin samples from foot and chest were used as controls.

A

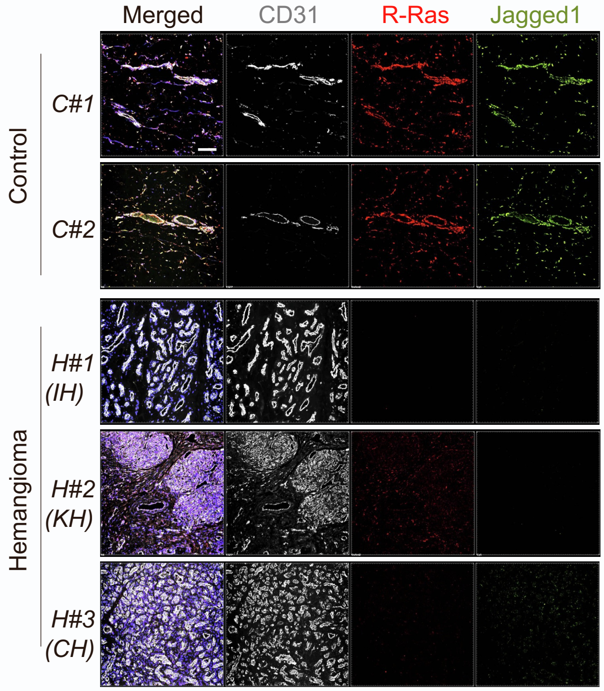

B

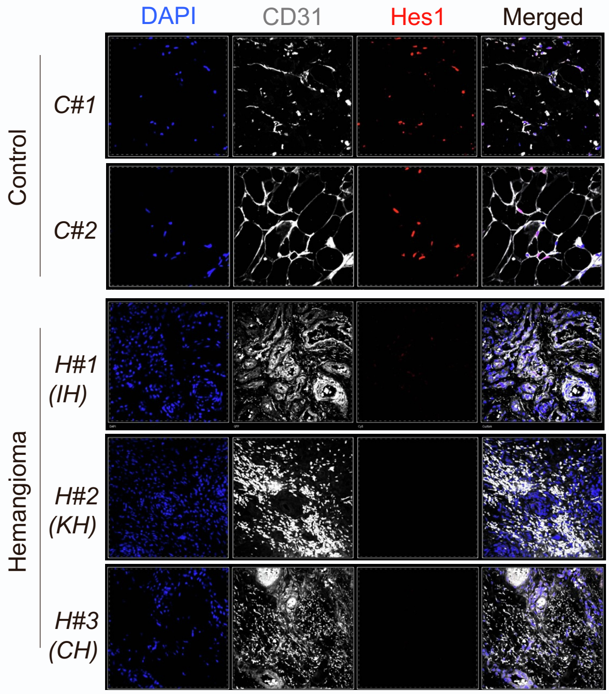

Supplementary Figure 12: Downregulation of R-Ras, Jagged1 and Hes1 in hemangioma and hemangioendothelioma.

a) CD31, R-Ras and Jagged1 immunofluorescence staining of pediatric skin control or hemangioma samples. R-Ras and Jagged1 are strongly downregulated in all samples analyzed from three patients with hemangioma, compared to control skin samples. C#1: pediatric skin from foot; C#2: pediatric skin from left chest; H#1: infantile hemangioma (IH; skin and subcutaneous tissue from posterior neck, 5-year-old male); H#2: kaposiform hemangioendothelioma (KH; skin, left forearm, 2-week-old male); H#3: infantile capillary hemangioma of face (CH; skin, forehead lesion, male, 1 year old). Scale bar, 100 μm

b) Similar analysis of Hes1 expression.

siRNA OLIGONUCLEOTIDES	SOURCE	IDENTIFIER
siRNA targeting *AKT1*	Millipore, Sigma	siHK0094
siRNA targeting *AKT2*	Millipore, Sigma	siHK0099
siRNA targeting *AKT3*	Millipore, Sigma	siHK0100
siRNA targeting *AKT3*	Ambion, Life Technologies	s19428
siRNA Universal Negative Control No.1	Millipore, Sigma	siC001
siRNA targeting *JAG1*	Ambion, Life Technologies	s1175
siRNA targeting *NOTCH1*	Ambion, Life Technologies	s453558
siRNA targeting *NOTCH3*	Ambion, Life Technologies	s9640
siRNA targeting *UNC5B*	Ambion, Life Technologies	s47701
siRNA targeting *RRAS*	Ambion, Life Technologies	4390827
siRNA Select Negative Control No.2	Ambion, Life Technologies	4390846

Supplementary Table 1: siRNA oligonucleotides used for gene silencing. Related to STAR Methods.

Target gene	Forward primer	Reverse primer
AKT1	5' GTT TTT GGG CTT GCG CTG GA	5' TCC CCA GAC TAG GAA AGC AAA G
AKT2	5' AAA GAA GGC TGG CTC CAC AA	5' GTC GCT CTT CAG CAG GAA GT
AKT3	5' CTC ACT GAA AGG GGG CAT GT	5' GCT CCT AGC ACC AAA GGG TT
NOTCH1 Duplex 1	5' CCA CCC CTC CTA GTT TGG GA	5' CCT CAC TGG CAT GAC ACA CA
NOTCH1 Duplex 2	5' TTG TTA GCC CCG TTC TTC AG	5' GTC AAC GCC GTA GAT GAC C
NOTCH2	5' CCC AAT GGG CAA GAA GTC TA	5' CAC AAT GTG GTG GTG GGA TA
NOTCH3	5' TGA GAC GCT CGT CAG TTC TT	5' TGG AAT GCA GTG AAG TGA GG
NOTCH4	Human Notch4 qPCR Primer Pair Cat: HP104650 SinoBiological (USA)	
JAG1	5' TCA CGG GAA GTG CAA GAG TC	5' TGC AAG TGC CAC CGT TTC TA
JAG2	5' GCG ATC TCT CCA CTG TGC TT	5' GTG GAC AGT TCC GAG GGT TC
DLL4	5' GGG CAC CTA CTG TGA ACT CC	5' CCA TTC TCC AGG TCA TGG CA
TP53	5' CCT ATG GAA ACT ACT TCC TGA AAA	5' TCC GGG GAC AGC ATC AAA TC
CDKN1A	5' GCA GAC CAG CAT GAC AGA TTT	5' GGA TTA GGG CTT CCT CCT GGA
HEY1	5' TGG ATC ACC TGA AAA TGC TG	5' TTG TTG AGA TGC GAA ACC AG
CDH5	5' GTC CTG CAG ATC TCC GCA AT	5' TGT TGG CCG TGT TAT CGT GA
18S	5' TGT GCC GCT AGA GGT GAA ATT	5' TGG CAA ATG CTT TCG CTT T

Supplementary Table 2: qPCR primers used for gene expression studies. Related to STAR Methods.
